# Disability related disparities in diagnosis and survival of digestive system cancers in Northern Italy

**DOI:** 10.1038/s41467-026-78262-w

**Published:** 2026-10-10

**Authors:** Flavia Pennisi, Carlotta Buzzoni, Federico Gervasi, Maria Teresa Greco, Carlo Signorelli, Antonio Giampiero Russo, Cristina Renzi

**Affiliations:** 1 School of Medicine, University Vita-Salute San Raffaele, Milan, Italy; 2 PhD National Programme in One Health approaches to infectious diseases and life science research, Department of Public Health, Experimental and Forensic Medicine, University of Pavia, Pavia, Italy; 3 Epidemiology Unit, Agency for Health Protection of Milan, Milan, Italy; 4 Research Department of Behavioural Science and Health, University College London, London, UK

**Keywords:** Cancer epidemiology, Risk factors, Colon cancer

## Abstract

Individuals with disabilities may experience disparities in cancer diagnosis, but population-based evidence remains limited. We conduct a population-based cohort study using linked cancer registry and administrative healthcare data for digestive system cancers diagnosed between 2018 and 2021 in Northern Italy, examining diagnostic routes and one-year survival according to pre-existing impairments. Individuals with impairments are more likely to be diagnosed following emergency presentation compared with those without impairments (51.8% compared with 37.9%; adjusted odds ratio 1.41, 95% confidence interval 1.23–1.61), particularly those with impairments of mental and neuromusculoskeletal functions. They also have higher one-year mortality (hazard ratio 1.36, 95% confidence interval 1.26–1.46), independent of socio-demographic and clinical factors. Here, we show that disability-related disparities in emergency cancer diagnosis and survival persist across multiple impairment categories, providing population-based evidence from a European setting on differences in diagnostic pathways across heterogeneous disability groups.

## Abstract (web summary)

Disparities in cancer diagnosis amongst patients with disabilities remains a challenge, yet there are limited European population-based studies addressing such issue. Here, the authors show that in Northern Italy, people with disabilities, particularly those with mental and neuromuscular impairments are more likely to be diagnosed with non-metastatic cancers following emergency presentation and also have a higher one-year mortality rate compared to those without impairments.

## Introduction

Cancer remains one of the leading causes of morbidity and mortality globally^1,2^. Although advancements in early detection and treatment have led to improved survival rates, substantial disparities persist across different population groups^3–5^. Individuals with disabilities represent a growing, yet historically underrepresented, population in cancer research and care. Globally, more than 1.3 billion people, approximately 16% of the world’s population, live with some form of disability^6,7^. In Italy, approximately one in four people has a disability, with mobility impairments (52%), hearing impairments (45%), and vision impairments (39%) being the most common categories^8^. In the Lombardy region, one of Italy’s most populous and demographically aged areas, disabilities affect one in five people, and nearly half of those aged 60 years and older (the age group most affected by cancer) report at least one disability^4,9–11^.

Emerging evidence suggests that people with disabilities may face a disproportionate cancer burden, both in terms of incidence and outcomes^12^. Despite national efforts toward inclusive healthcare^13–15^, people with disabilities continue to face substantial challenges in accessing timely and appropriate medical services, including cancer diagnosis and treatment^16^. Individuals with disabilities may encounter multiple barriers across the cancer diagnostic pathway, including reduced access to screening, difficulties in recognising or communicating symptoms, physical or organisational barriers to accessing diagnostic testing, and the potential for diagnostic overshadowing in clinical settings^17,18^. These factors may increase the likelihood of emergency presentation (EP) and, consequently, may adversely affect cancer prognosis and survival^19^.

A recent UK study found that individuals with mental health conditions presenting with red-flag symptoms of colorectal cancer were 28% less likely to undergo timely colonoscopy and more likely to be diagnosed via EP. The recent COVID-19 pandemic has highlighted and further amplified pre-existing barriers^20^. In Lombardy, one of the first European regions affected by the COVID-19 pandemic, the healthcare system experienced strain. The reorganisation of services and disruption of routine care substantially affected access to diagnostic pathways^21^.

Despite the growing recognition of these challenges, there is a scarcity of population-based studies investigating disability-related disparities in cancer diagnosis and outcomes, especially in the European context. Existing evidence largely focuses on screening uptake, while little is known about other diagnostic routes, including emergency and in- and outpatient routes to cancer diagnosis among symptomatic patients with disabilities^22^. Moreover, previous epidemiological studies have primarily focused on individuals with intellectual or learning disabilities^23^, with no population-based evidence available for other impairment domains, and have often examined single conditions rather than capturing the heterogeneity of impairments within a common analytical framework^24^. Understanding whether individuals with disabilities experience different routes to cancer diagnosis is therefore essential to identify potential inequalities in access to timely diagnostic care. In Italy, organised population-based screening programmes are available for colorectal cancer and are based on biennial faecal immunochemical testing (FIT)^25^. In the Lombardy region, screening invitations are offered to individuals aged 50–74 years^26^; however, participation remains suboptimal (approximately 42–45%)^27^. No organised population-based screening programmes are currently available for other digestive system cancers included in this study, such as pancreatic, hepatobiliary, oesophageal or gastric cancers.

In this work, we examine disability-related disparities in diagnostic routes and survival for digestive system cancers within a population-based cohort in Northern Italy, using individually linked data from a population-based cancer registry and administrative health databases. We evaluate diagnostic routes and one-year survival according to disability status, operationalised through International Classification of Functioning, Disability, and Health (ICF)-based impairment categories^28^, while accounting for cancer and patient characteristics. We show that individuals with pre-existing impairments are more likely to be diagnosed following emergency presentation and have lower one-year survival than individuals without impairments, particularly among those with mental and neuromusculoskeletal impairments. These findings provide population-based evidence from a European setting that disability-related inequalities persist across multiple impairment categories and affect both cancer diagnostic pathways and early survival.

## Results

### Participant characteristics

Between 2018 and 2021, out of all digestive system cancers, 8589 individuals (50.6%) were diagnosed with colorectal cancer, 5364 (31.6%) with hepatopancreatobiliary (HPB) cancer, and 3,011 (17.7%) with upper gastrointestinal (GI) cancer in the provinces of Milan and Lodi. Among all cancer patients, 19.0% (*n* = 3222) had at least one pre-existing impairment. Specifically, 6.5% of individuals had documented impairments of mental functions (*n* = 1110), 2.8% of sensory functions (*n* = 470), 3.1% of neuromusculoskeletal and movement-related functions (*n* = 525), 5.8% of continence functions (*n* = 976), and 10.2% impairments related to prosthetic or orthotic use (*n* = 1736) (Supplementary Dataset 1). The median age was 81 years for individuals with impairments of sensory and continence functions and functions related to prosthetic or orthotic use; 82 years for those with impairments of mental functions; 78 years for those with impairments of neuromusculoskeletal and movement-related functions; 73 years for those with any impairments; 74 years for those with no impairments.

Multiple impairments (≥2 concurrent body function impairments or use of assistive devices) were observed in 1380 individuals (8.1%). Specifically, 5.3% had two impairments and 2.8% had three or more. The most frequent combinations were impairments of sensory functions with impairments related to prosthetic or orthotic use (1.3%) and impairments of continence functions with impairments related to prosthetic or orthotic use (1.0%).

People with impairments, compared with those without, were more frequently socioeconomically disadvantaged, had a higher prevalence of two or more chronic conditions, and were less likely to have metastatic cancer at diagnosis. EP was significantly more frequent among individuals with impairments than among those without, ranging from 46.1% in those with impairments of sensory functions to 51.8% in those with impairments of mental functions, compared with 36.8% among individuals without impairments (Supplementary Dataset 1).

EP was consistently more frequent among patients with impairments across all cancer types: 44.7% vs 32.2% in colorectal cancer, 49.3% vs 42.4% in HPB cancer, and 53.9% vs 39.4% in upper gastrointestinal cancer. Overall, this pattern persisted when considering all cancers combined (47.6% vs 36.8%) (Supplementary Table 1)

### Multivariable analyses estimating the likelihood of EP by impairments and patient characteristics

The presence of any pre-existing impairment versus no impairment was associated with a significantly higher likelihood of EP at multivariable analysis (aOR = 1.39, 95%CI 1.29–1.50). In analyses of specific impairment categories (Supplementary Dataset 2 and Supplementary Fig. 1), results were consistent across sequential models. In the age- and sex-adjusted model (Supplementary Dataset 2, Model 1), impairments of mental functions, neuromusculoskeletal and movement-related functions, and impairments related to prosthetic or orthotic use were associated with higher odds of EP compared with no impairment. These associations remained stable after further adjustment for socioeconomic factors and comorbidity burden (Supplementary Dataset 2, Model 2) and after inclusion of cancer-related characteristics (Supplementary Dataset 2, Model 3). In the fully adjusted model (Model 3), impairments of mental functions (aOR=1.41, 95% CI 1.23–1.61), neuromusculoskeletal and movement-related functions (aOR=1.30, 95% CI 1.08–1.57), and impairments related to prosthetic or orthotic use (aOR=1.24, 95% CI 1.10–1.40) were associated with higher odds of EP. The odds of EP were also higher for metastatic versus non-metastatic disease at diagnosis (aOR=1.81, 95% CI 1.68–1.96), for HPB (aOR=1.40, 95% CI 1.30–1.50) and upper GI (aOR=1.31, 95% CI 1.20–1.43) compared with colorectal cancer, as well as for patients aged <50 or ≥70 years, those in the most deprived socioeconomic categories, and individuals with ≥3 chronic conditions.

Additionally, the COVID-19 versus the pre-pandemic period was also associated with a higher likelihood of cancer being diagnosed following EP (aOR=1.18, 95% CI 1.12–1.25). We did not find evidence of interactions between impairment status and period (pre-pandemic versus pandemic). In period-stratified models, findings were consistent, with somewhat stronger associations during the COVID-19 period for impairments of mental functions (aOR=1.44, 95% CI 1.19–1.75) and impairments related to prosthetic or orthotic use (aOR=1.32, 95% CI 1.11–1.58) (Supplementary Table 2). We did not find evidence of interactions between impairment status and tumour site.

### Overall survival stratified by impairment status, cancer metastatic status and diagnostic route

**Fig. 1:**
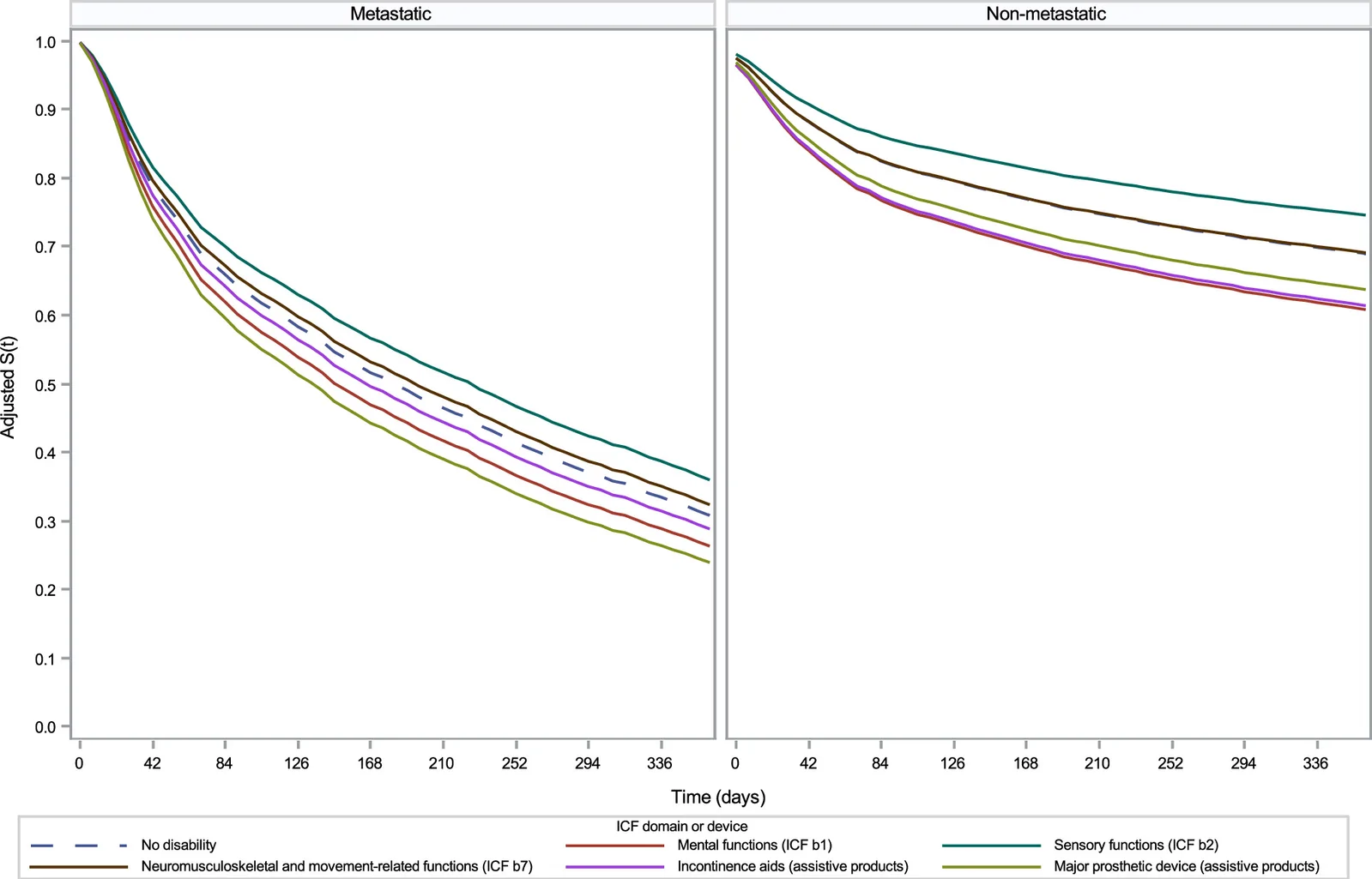
Adjusted one-year survival curves stratified by metastatic and non-metastatic status at diagnosis. Adjusted one-year survival curves are shown for patients with digestive system cancers diagnosed in 2018–2021 in the provinces of Milan and Lodi, northern Italy (*n* = 16,964), stratified by metastatic and non-metastatic status at diagnosis and by ICF-based impairment categories (mental functions, sensory functions and pain, neuromusculoskeletal and movement-related functions, continence functions, and prosthetic or orthotic use), compared with patients without impairments. Models were adjusted for cancer site, sex, age group, number of chronic conditions, diagnostic route (emergency presentation vs. screening/inpatient/outpatient visits), and period (pre- vs. during COVID-19). Adjusted survival curves are derived from multivariable Cox proportional hazards regression models and estimated using direct standardisation over the observed joint distribution of the remaining adjustment covariates in the study population. Hazard ratios with 95% confidence intervals are reported in Supplementary Table 3. Wald tests for hazard ratios are two-sided. No adjustment was made for multiple comparisons.

In analyses stratified by metastatic status at diagnosis, patients with metastatic disease exhibited a poor overall prognosis. Following multivariable adjustment, the presence of a major prosthetic device was associated with a significantly increased hazard of death at one year (HR = 1.27, 95% CI 1.10–1.46) (Fig. 1 and Supplementary Table 3). By contrast, in patients with non-metastatic disease, marked impairment-related disparities in survival were observed. Impairments of mental functions were associated with a higher risk of death at one year (HR = 1.42, 95% CI 1.30–1.54), as were impairments related to continence aids (HR = 1.39, 95% CI 1.26–1.52) and major prosthetic devices (HR = 1.26, 95% CI 1.16–1.37). Conversely, impairments of sensory functions were associated with a lower mortality risk at one year (HR = 0.76, 95% CI 0.66–0.87).

**Fig. 2:**
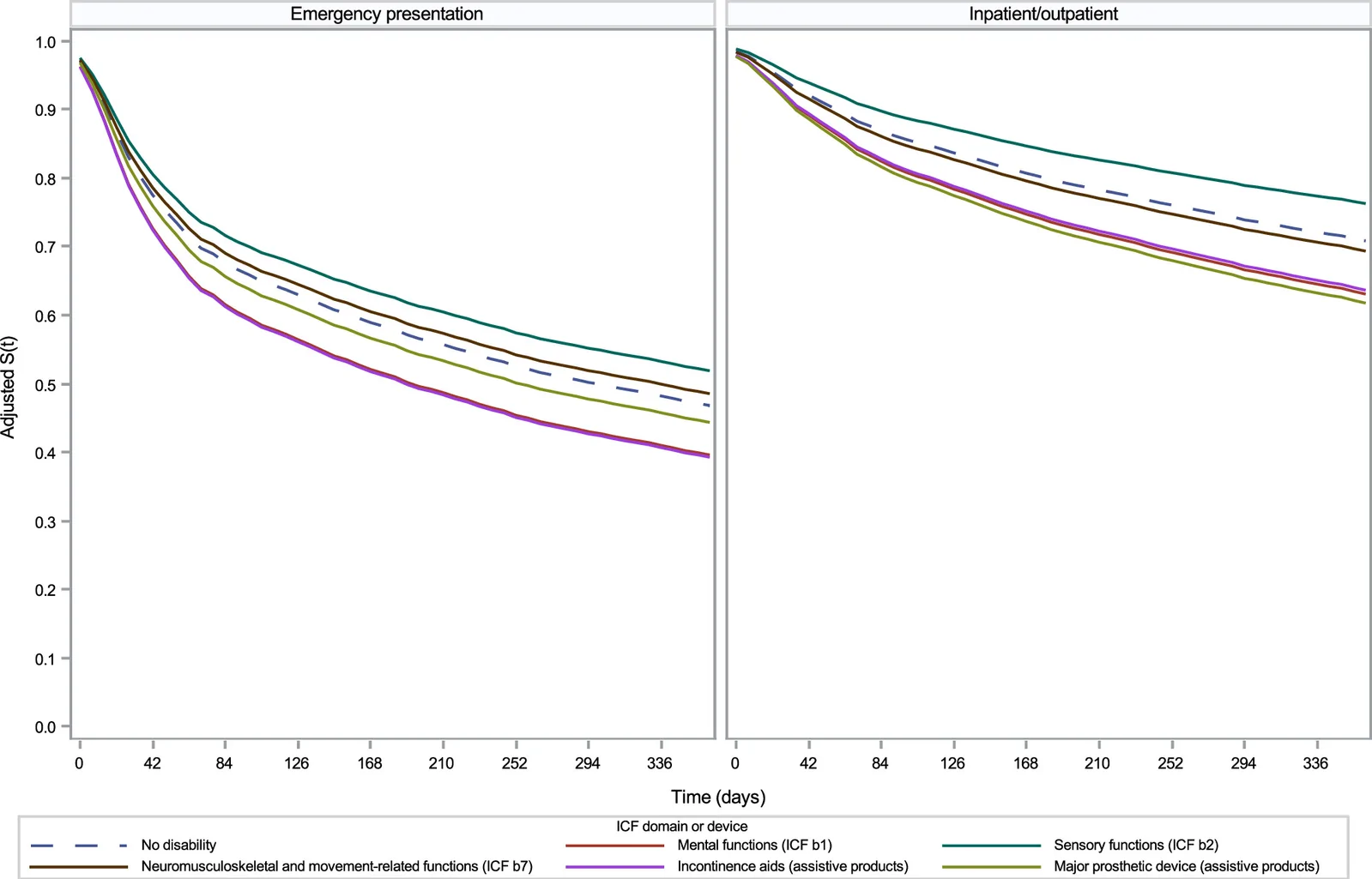
Adjusted one-year survival curves stratified by diagnostic route. Adjusted one-year survival curves are shown for patients with digestive system cancers diagnosed in 2018–2021 in the provinces of Milan and Lodi, northern Italy (*n* = 16,964), stratified by emergency presentation and non-emergency diagnostic routes and by ICF-based impairment categories (mental functions, sensory functions and pain, neuromusculoskeletal and movement-related functions, continence functions, and prosthetic or orthotic use), compared with patients without impairments. Models were adjusted for cancer site, sex, age group, number of chronic conditions, period (pre- vs. during COVID-19), and metastatic status at diagnosis. Adjusted survival curves are derived from multivariable Cox proportional hazards regression models and estimated using direct standardisation over the observed joint distribution of the remaining adjustment covariates in the study population. Hazard ratios with 95% confidence intervals are reported in Supplementary Table 4. Wald tests for hazard ratios are two-sided. No adjustment was made for multiple comparisons.

Disparities by impairment status also emerged when examining survival by diagnostic route, accounting for potential confounding factors (Fig. 2 and Supplementary Table 4). Among patients diagnosed through the non-EP routes, impairments related to prosthetic or orthotic use (HR = 1.50, 95% CI 1.36–1.67) were associated with a higher hazard of death at one year compared to patients with no impairment, as were impairments of mental functions (HR = 1.42, 95% CI 1.27–1.59) and impairments of continence functions (HR = 1.39, 95% CI 1.23–1.56). Impairments of sensory functions were associated with a lower hazard of death (HR = 0.75, 95% CI 0.63–0.90). Among patients diagnosed following EP, compared to the non-EP route, the overall prognosis was poorer. In this group, impairments of mental functions (HR = 1.28, 95% CI 1.16–1.42) and impairments of continence functions (HR = 1.30, 95% CI 1.15–1.45) were significantly associated with an increased hazard of death at one year compared with individuals without such impairments. Impairments of sensory functions were associated with a borderline reduction in mortality risk (HR = 0.84, 95% CI 0.71–1.00).

We found evidence of interaction between tumour site and impairment status for mental functions, continence functions, and major prosthetic device use in relation to OS. When survival analysis was stratified by tumour site and metastatic status (Supplementary Fig. 2), marked differences in survival were observed across tumour sites. However, among non-metastatic patients, curves consistently indicated lower survival for individuals with impairments related to mental functions, continence aids, and major prosthetic device use. These patterns were supported by the corresponding HRs (Supplementary Table 5 and Supplementary Table 6). Mental function impairments were associated with higher mortality in colorectal (HR = 1.63, 95% CI 1.37–1.93), upper GI (HR = 1.47, 95% CI 1.11–1.96), and HPB cancers (HR = 1.28, 95% CI 1.06–1.54). Similar associations were observed for continence aids (colorectal: HR = 1.28, 95% CI 1.08–1.51; upper GI: HR = 1.35, 95% CI 0.99–1.83; HPB: HR = 1.62, 95% CI 1.31–1.99) and major prosthetic device use (colorectal: HR = 1.45, 95% CI 1.22–1.71; upper GI: HR = 1.59, 95% CI 1.25–2.03; HPB: HR = 1.45, 95% CI 1.23–1.70).

When stratifying by diagnostic route (Supplementary Fig. 3), distinct patterns emerged in both the adjusted survival curves and the regression estimates. Among patients diagnosed following emergency presentation (EP), survival curves consistently showed poorer outcomes for individuals with impairments of mental functions and continence across tumour sites. This was reflected in increased mortality risks for mental function impairments (HR = 1.37, 95% CI 1.17–1.61 in colorectal; HR = 1.48, 95% CI 1.18–1.86 in upper GI; HR = 1.09, 95% CI 0.91–1.29 in HPB cancers) and continence-related impairments (HR = 1.29, 95% CI 1.09–1.51; HR = 1.37, 95% CI 1.05–1.79; HR = 1.27, 95% CI 1.04–1.57, respectively), whereas associations for major prosthetic device use were less consistent.

In contrast, among patients diagnosed through non-EP routes, survival curves showed a broader disadvantage across impairment categories. This pattern was supported by the hazard ratios, with consistently higher mortality observed for mental function impairments in colorectal (HR = 1.63, 95% CI 1.37–1.93), upper GI (HR = 1.47, 95% CI 1.11–1.96), and HPB cancers (HR = 1.28, 95% CI 1.06–1.54). Similar patterns were observed for continence aids (colorectal: HR = 1.28, 95% CI 1.08–1.51; upper GI: HR = 1.35, 95% CI 0.99–1.83; HPB: HR = 1.62, 95% CI 1.31–1.99) and major prosthetic device use (colorectal: HR = 1.45, 95% CI 1.22–1.71; upper GI: HR = 1.59, 95% CI 1.25–2.03; HPB: HR = 1.45, 95% CI 1.23–1.70). In contrast, no consistent survival disadvantage was observed for sensory impairments, in line with survival curves showing overlapping or slightly improved survival in some groups (e.g., colorectal cancer: HR = 0.69, 95% CI 0.52–0.90).

## Discussion

Reducing emergency cancer diagnoses has been recognised as a key public health priority, yet little is known about how they affect people with disabilities. In this population-based study, individuals with pre-existing impairments were more likely to be diagnosed with cancer of the digestive system following an EP and had a poorer prognosis compared with individuals without pre-existing impairments, independently of socio-demographic and cancer characteristics. These disparities were particularly evident for individuals with impairments of mental and continence functions, as well as for those with impairments related to prosthetic or orthotic use, which also represented the most prevalent impairment categories in the study population. Specific impairment categories were independently associated with a higher risk of death at one year, in both metastatic and non-metastatic cancers.

Population-based evidence on cancer diagnostic routes and health outcomes for people with pre-existing disabilities is scant, despite the demographic transition towards ageing populations, paralleled by an increase in disability prevalence^29,30^. This study provides population-based evidence from a European setting on disparities in emergency cancer diagnosis across multiple impairment categories. Few large-scale investigations have addressed cancer care disparities in individuals with specific disabilities^31^, with most studies relying on qualitative methods and small samples^24,32^. There is particularly scant evidence from Southern and Eastern Europe and on non-cognitive functional impairments^33–39^.

The limited available studies consistently report poorer prognosis^23,34,36,39–41^, lower access to guideline-concordant therapies^37,40,42–44^, and marked care disparities^38,42^, but data on diagnostic pathways remain scarce. While some studies^18^ have examined participation of people with disabilities in cancer screening programmes, only one population-based investigation has evaluated the risk of EP among adults with intellectual disabilities. In that study, Heslop et al.^45^. observed that 35% of cancers among deceased adults with intellectual disabilities were diagnosed through EP in England. However, unlike in our study, the authors did not provide a comparator group from the general population to quantify whether this proportion was higher than expected. By focusing on EP as a route to diagnosis, our study addresses a relevant and underexplored dimension of cancer inequalities among people with disabilities.

The current study highlighted the greatest EP risk among individuals with impairments of mental functions. Diagnostic overshadowing, fragmented care, stigma, and communication barriers may all play a role in these individuals, with delays in help-seeking for possible cancer symptoms and/or delays in referral for diagnostic investigations^4,46,47^. Our findings are in line with previous studies, where patients with dementia were more likely to present with cancer when acute complications occur (such as bowel obstruction or perforation for GI cancer) and are also more likely to have cancer diagnosed as an incidental finding or after death^48^. Except for advanced age, dementia was identified as the most significant individual risk factor for EP in England^49^, surpassing several other comorbidities and sociodemographic factors.

These mechanisms should also be interpreted in the context of differences in screening availability across cancer sites. In Italy, organised population-based screening programmes are implemented for colorectal cancer, whereas no screening programmes currently exist for the other digestive system cancers included in this study. Previous studies have reported lower participation in cancer screening programmes among people with disabilities, suggesting that inequalities in screening uptake may also contribute to the disparities observed for colorectal cancer^50,51^. Notably, even for cancers where screening is available, such as colorectal cancer, most cases are still diagnosed following symptomatic presentation rather than through screening programmes, supporting our focus on emergency presentation as a key indicator of inequalities in diagnostic pathways^5^.

Among individuals with impairments of continence functions or impairments related to prosthetic/orthotic use, plausible contributors to increased risk of EP and poorer survival include mobility restrictions, high dependence on caregivers, and high levels of multimorbidity. Healthcare facilities often exacerbate barriers due to inadequate physical accessibility, because of a lack of ramps, narrow doorways, or non-adjustable examination tables, including imaging devices unsuitable for individuals with mobility impairments, hindering effective diagnostic and care procedures^16,52^. Healthcare providers frequently lack training and awareness about the specific needs of individuals with physical impairments, resulting in fragmented care and failure to provide appropriate accommodations^32^.

Individuals with impairments of sensory functions remain a comparatively underexplored group in the context of cancer diagnostic pathways, despite facing specific challenges^53^. Unlike physical disabilities, they are more likely to encounter barriers related to communication and access to information due to the limited availability of accessible materials (e.g., braille resources, sign language interpretation), compounded by insufficient training of healthcare professionals^54^. However, our findings did not show a higher risk of EP or worse cancer outcomes in this group. Sensory impairments such as vision or hearing loss are relatively common in older populations and may not necessarily reflect severe underlying health deterioration compared with other impairment categories. Differences in healthcare engagement and support structures may also contribute. Individuals with sensory impairments may have more frequent contact with healthcare services due to ongoing monitoring or management of sensory conditions, potentially facilitating earlier symptom recognition and diagnostic evaluation^55^. In addition, they may more often attend healthcare encounters with accompanying persons who support communication and care navigation. Previous evidence suggests that accompaniment to physician visits is substantially more common among individuals with sensory impairments and may improve care coordination and adherence to planned diagnostic pathways^56,57^. Nevertheless, variability in impairment severity and residual confounding cannot be excluded.

Although our findings indicated that individuals with impairments had a higher likelihood of non-metastatic cancer at diagnosis, possibly reflecting more frequent healthcare contacts, they were disproportionately diagnosed through EP. In line with previous evidence^58,59^, this diagnostic route is associated with lower survival, independently of stage at diagnosis. This suggests that differences in survival are not fully explained by disease stage at diagnosis and may reflect inequalities in access to timely diagnostic and care pathways. Our findings highlight survival disparities, especially for people with impairments of mental functions, continence or prosthetic/orthotic impairments diagnosed with non-metastatic cancer, suggesting sub-optimal access to appropriate treatment^33,60,61^. These findings are consistent with the interpretation that impairment-related barriers may have a greater impact in potentially treatable diseases, where timely diagnosis and management are critical for prognosis. When examining survival curves stratified by tumour site, disability-related inequalities in survival appeared more pronounced for colorectal cancer, whereas differences were less marked for tumours with poorer prognosis, including hepatobiliary and upper gastrointestinal tumours. This pattern suggests opportunities for reducing inequalities and improving early diagnosis and outcomes, especially for those cancers for which we currently have very good diagnostic and treatment options. These findings suggest that disability status should be explicitly considered when designing strategies aimed at reducing emergency cancer diagnoses, including initiatives to improve timely symptom recognition and access to diagnostic investigations in vulnerable populations.

We observed a higher risk of EP during the COVID-19 period, consistent with disruptions to elective care and diagnostic services^62^. In addition, changes in healthcare-seeking behaviour and reduced opportunities for early symptom assessment may have contributed to delays in cancer diagnosis during this period. Although no strong interactions between disability and pandemic period were detected, our estimates suggested a slightly increased vulnerability during COVID-19 for individuals with impairments of mental functions or prosthetic/orthotic impairments. This finding highlights how systemic crises may exacerbate pre-existing inequities and underscores the importance of safeguarding continuity of cancer care for vulnerable groups during future health system shocks.

The consistently higher risk of EP and survival disadvantage for individuals with specific impairment categories, especially for non-metastatic cancers and accounting for comorbidities, suggests that disability, as captured through impairment categories, may represent an independent predictor of sub-optimal diagnostic pathways associated with poorer health outcomes. Reducing emergency cancer diagnosis should be a key priority, as EPs do not allow for appropriate planning of healthcare, both for the affected individual and for the wider community. Interventions at multiple levels are warranted, including improving accessibility of preventive and diagnostic services, strengthening integration between oncology and disability care, and developing targeted outreach strategies to ensure timely recognition of symptoms and referral.

At the clinical level, healthcare professionals should be supported with targeted training and practical tools to improve recognition of potential cancer symptoms in people with disabilities, including the use of structured symptom assessment approaches and clinical decision support, to reduce diagnostic overshadowing^63^.

At the health system level, screening programmes and diagnostic services should ensure that invitations and information materials are provided in accessible formats (e.g., easy-read versions, large print, braille or digital formats compatible with assistive technologies), alongside physically accessible clinical environments and facilitation of caregiver or support person involvement during healthcare encounters^64^. Patient navigation programmes and improved coordination between primary care, oncology services and disability support services may further facilitate timely referral and diagnostic assessment^65^.

From a policy perspective, incorporating disability indicators into routine cancer monitoring may help identify vulnerable groups at higher risk of suboptimal diagnostic pathways and support the development of targeted interventions aimed at improving equity in cancer care. Given the ageing of the population and the increasing prevalence of disability and multimorbidity, strengthening disability-inclusive cancer services will be essential to ensure equitable access to timely diagnosis and treatment.

We developed a methodology for classifying cancer diagnostic routes using linked cancer registry and administrative health data, which is transferable to other regional and national health systems. Similar to the English Routes to Diagnosis algorithm^66^, this approach leverages routinely collected, population-based datasets to enhance cancer surveillance and identify areas for improvement. The use of high-quality cancer registry data underpins the reliability and validity of our findings. The application of an ICF-based framework represents a key strength, as it enables the systematic and comparable assessment of multiple impairment domains beyond traditional diagnosis-based classifications. The large sample size ensured robust statistical power for performing stratified analyses by metastasis status and diagnostic route.

Some limitations should be acknowledged. Our disability definition, based on administrative indicators of impairment mapped to ICF categories, may not fully capture functional status, the severity of functional limitations, or psychosocial dimensions of disability. As a result, the indicators used in this study should be interpreted as proxies for underlying functional impairments rather than direct measures of disability. In addition, for some impairment domains, such as continence functions, residual misclassification cannot be excluded, as these indicators may in some cases reflect manifestations of advanced disease rather than pre-existing disability, particularly in colorectal cancer. The impairment groupings used in this study reflect the ICF-based classification available in the administrative datasets and may not fully correspond to other commonly used disability typologies. The mechanisms underlying the observed associations could not be directly assessed, and further studies are needed to investigate the pathways linking impairment status to diagnostic routes and outcomes. Finally, unmeasured confounding from lifestyle factors (e.g., smoking, alcohol), social support, or health literacy cannot be excluded.

This population-based study provides evidence from a European setting on disparities in cancer diagnosis and outcomes among people with disabilities, particularly among individuals with impairments of mental functions and those with continence or prosthetic-related impairments. These inequalities are particularly marked in non-metastatic cancers and persist even after accounting for comorbidities, suggesting that disability-related impairments may act as an independent predictor of sub-optimal diagnostic pathways associated with poorer health outcomes. Given the shift towards ageing populations, paralleled by an increase in disability and multimorbidity, appropriate integrated health services are essential to improve patient-centred and equitable cancer care.

## Methods

### Study design and study population

This study complied with all relevant ethical regulations. The study protocol was approved by the Comitato Etico Territoriale Lombardia 3, approval number 5328_20.11.2024_P. The requirement for informed consent was waived by the Comitato Etico Territoriale Lombardia 3 because the study was based on anonymised routinely collected cancer registry and administrative healthcare data and involved no direct contact with participants. Participants did not receive compensation.

A retrospective, population-based cohort study was conducted examining data from the Milan Cancer Registry, linked to administrative health databases from the Agency for Health Protection of Milan (ATS Milan), which is part of the Lombardy Healthcare System, northern Italy. The study area includes the provinces of Milan and Lodi, comprising approximately 3.5 million residents. Deterministic record linkage was performed using anonymised individual health-system beneficiary codes. The cohort included upper gastrointestinal (GI) (oesophagus and stomach), colorectal and hepatopancreatobiliary (HPB) malignant cancers based on the ICD-O-3.2 Classification World Health Organization (2019) and diagnosed in 2018-2021 among residents of the ATS Milan area. The study population included 16,964 patients, of whom 9,490 were men and 7,474 were women. The study followed the Strengthening the Reporting of Observational Studies in Epidemiology (STROBE) reporting guidelines.

### Data sources and variable definitions

The main outcome variables were ‘Route to diagnosis’ (RtD) and one-year survival. RtD refers to the sequence of interactions between a patient and the healthcare system leading to a cancer diagnosis, in line with previous UK and international studies. We developed an algorithm to infer the RtD for each study subject based on healthcare contacts in the 6 months preceding cancer diagnosis. Details of the algorithm have been previously described. RtD were classified into three mutually exclusive categories: (1) screening, (2) EP and (3) inpatient/outpatient visits (IP/OP).

The main explanatory variable was the presence of a pre-existing impairment, defined using a validated algorithm developed by the ATS Milan, which integrates electronic health and social services databases. In the first step, diagnostic codes from social and healthcare flows (including exemptions from the copayment fee for health services, rehabilitation services, nursing home care, residential/semi-residential facilities and domiciliary care) were screened and mapped to the ICF. The ICF provides an internationally standardised framework for classifying functioning and disability, conceptualising disability as a multidimensional construct encompassing impairments, activity limitations and participation restrictions within a biopsychosocial model of health. This approach allowed us to operationalise pre-existing functional impairments within a standardised conceptual framework. In a second step, healthcare flows (hospital discharges, outpatient specialist visits), specific flows associated with financial support provided by the Lombardy Region to people with disabilities and registries of medical devices were interrogated to capture additional cases, distinguishing between incontinence-related devices and major prosthetic or orthotic use (e.g., motor or limb prostheses). The use of linked administrative health and social care data allows the identification of large populations with functional impairments in population-based studies when direct measures of functioning are not available. Each individual was classified according to the structure and function affected by the impairment into one of 8 ICF-based impairment categories: Mental, Sensory and Pain, Phonatory and Speech Production, Cardiovascular, Respiratory, Haematological, and Immune Systems, Digestive, Metabolic, and Endocrine Systems, Genitourinary and Reproductive, Neuromusculoskeletal and Movement-Related, Skin and Related Structures. In cases where it was not possible to classify the impairment according to the ICF, but the individuals appeared in the prosthetics data flows or were beneficiaries of disability exemptions with attendance allowance, three categories were created: major prosthetic or orthotic use, the presence of a disability exemption with attendance allowance, and the presence of incontinence-related devices. The methodological details of the algorithm are reported in a previous paper. To reduce variability, only impairment categories with a prevalence ≥2% in the study sample were included in the analyses. For the study, we included 5 types of impairment classification: mental functions, sensory functions, neuromusculoskeletal and movement-related functions, continence functions, functions related to prosthetic or orthotic use and, finally, any impairment. Individuals with more than one impairment were classified in all corresponding impairment categories. To account for co-occurrence, regression models were adjusted for the presence of all impairment categories.

Additional explanatory variables included socio-demographic characteristics (age, sex, deprivation index, marital status and education level), cancer site, cancer metastatic status at diagnosis, number of physical comorbidities and period of cancer diagnosis (before and during/after the COVID-19 pandemic). Data on cancer site, date of diagnosis and metastatic status at diagnosis, as well as on sex and age, were obtained from the Milan Cancer Registry, which is part of the Italian Association of Cancer Registry (AIRTum) and the International Association of Cancer Registries (IACR). Sex was recorded in the cancer registry and administrative healthcare databases. Gender identity was not available in these data sources. Sex was considered in the study design and analysis as an a priori adjustment variable in all regression models; analyses stratified by sex were not performed because the primary aim was to examine disability-related disparities in diagnostic routes and survival. Patients’ socioeconomic level has been defined using the deprivation index calculated from Italian census data at the census section level, adopting the Rosano revised version of the Caranci index. The index considers 5 socioeconomic traits of the resident population and is divided into 5 levels corresponding to the quintiles of its distribution (1–5, 1 indicating the least deprived level and 5 the most deprived one). The number of physical comorbidities (0, 1, 2, or ≥3) was defined using the total number of physical chronic conditions per subject, according to the criteria and codes established by the 2017 Lombardy Region’s deliberation n° X/6164, linking health records up to 6 months pre-cancer diagnosis. Five non-mutually exclusive chronic condition categories were considered: type 2 diabetes and cardiovascular, neurological, genitourinary and cerebrovascular diseases.

### Statistics and reproducibility

No statistical method was used to predetermine sample size. The study included all eligible residents of the Agency for Health Protection of Milan area diagnosed with upper gastrointestinal, colorectal, or hepatopancreatobiliary malignant cancers between 2018 and 2021 and recorded in the Milan Cancer Registry. No data were excluded from the analyses after application of the predefined eligibility criteria. The study was observational and based on routinely collected data; the analyses were not randomised. The investigators were not blinded to exposure status, outcome status, or covariate information during data analysis and outcome assessment.

We employed descriptive statistics to examine baseline socio-demographic and clinical characteristics of individuals with different impairment categories. Characteristics of individuals with versus without impairments were compared using Pearson’s χ² test.

Logistic regression was applied to estimate crude and adjusted odds ratios (aORs) with 95% confidence intervals (CIs) for EP versus non-EP. Initially, a single binary exposure (any impairment vs none) was included in a multivariable model. Subsequently, the five impairment categories, modelled as binary indicators, were included simultaneously in a multivariable model. Based on previous evidence and clinical reasoning, potential confounders included the number of comorbidities, sociodemographic characteristics (age, sex, deprivation), cancer site, metastatic status (as an indicator of disease stage) and period of diagnosis (before or during the COVID-19 pandemic). Covariates were selected a priori to account for factors that may influence both disability status and access to timely cancer diagnosis, and therefore potentially confound the association between disability and diagnostic routes. Given the strong association between disability and age, age and sex were included as a priori covariates in all regression models to account for potential confounding. Regression models were adjusted sequentially to evaluate the impact of progressive adjustment: Model 1 included age and sex; Model 2 additionally included deprivation and number of chronic conditions; and Model 3 further adjusted for cancer-related variables (cancer site, metastatic status) and period of diagnosis. We formally tested for interaction between disability status and period (pre-pandemic vs. pandemic), as well as between impairment categories and cancer site, for both outcomes of interest, EP and survival. Interaction terms were introduced into the multivariable regression models and evaluated using likelihood ratio tests.

OS was defined as the time from diagnosis to death from any cause, with individuals censored at the end of follow-up if alive. We focused on one-year OS as the primary outcome, as short-term survival is more closely related to diagnostic pathways, including EP and is less influenced by longer-term disease progression and treatment effects. OS was analysed using multivariable Cox proportional hazards regression models including all five impairment categories simultaneously and adjusting for cancer site, age at diagnosis, sex, number of chronic comorbidities, metastatic status at diagnosis and RtD. Hazard ratios (HRs) with 95% CIs were estimated. To aid interpretation of the adjusted associations, covariate-adjusted survival curves were generated both for the overall study population and separately for each tumour site. Survival curves were estimated within analyses stratified by metastatic status at diagnosis and by RtD. Within each stratum, adjusted survival curves were derived from the fitted Cox proportional hazards models using direct standardisation over the observed joint distribution of all remaining adjustment covariates in the study population, thereby estimating adjusted survival probabilities over time. The proportional hazards assumption was assessed using Schoenfeld residuals.

Statistical significance was defined as a two-sided *p* value < 0.05. All analyses were conducted using SAS software (version 9.4; SAS Institute Inc., Cary, NC, USA).

### Consent and approval

The Ethics Committee has approved the study “Comitato Etico Territoriale Lombardia 3” (approval number 5328_20.11.2024_P).

### Reporting summary

Further information on research design is available in the Nature Portfolio Reporting Summary linked to this article.

## Supplementary information

Supplementary Information

Description of Additional Supplementary Files

Supplementary dataset 1

Supplementary dataset 2

Reporting Summary

Transparent Peer Review file

## Source data

Source Data

## Data Availability

The individual-level data used in this study are available under restricted access because they are derived from linked electronic health records, cancer registry data, and administrative healthcare databases held by the Agency for Health Protection of Milan (ATS Milano) and the Milan Cancer Registry, and contain sensitive patient health information. Access can be obtained only after approval by the relevant data custodians and ethics or governance bodies, in accordance with applicable regulations. Requests should be directed to the corresponding author, who will facilitate contact with the data custodians. Access cannot be guaranteed and, if approved, will be limited to the purposes and duration specified in the approved research protocol and data sharing agreement. The raw individual-level data are protected and are not publicly available due to data privacy laws. Summary results generated in this study are provided in the Article and Supplementary Information. The aggregate hazard ratio estimates and 95% confidence intervals underlying Figs. 1 and 2 and Supplementary Figs. 2 and 3 are provided in the corresponding source data files and in the Supplementary Information. Source data are provided with this paper.

## Code availability

The code used in this analysis is available via the GitHub repository: (https://github.com/FlaviaPennisi/digestive-cancer-disability-analysis-code).
