# Supplementary material for: Disability related disparities in diagnosis and survival of digestive system cancers in Northern Italy
Source: Nat Commun. 2026 Oct 10;17:11553. doi: 10.1038/s41467-026-78262-w (PMC13748588; doi:10.1038/s41467-026-78262-w)
Supplement: Supplementary file 1 [file 41467_2026_78262_MOESM1_ESM.pdf]

Disability related disparities in diagnosis and survival of digestive system cancers in Northern Italy

Supplementary Figure 1.

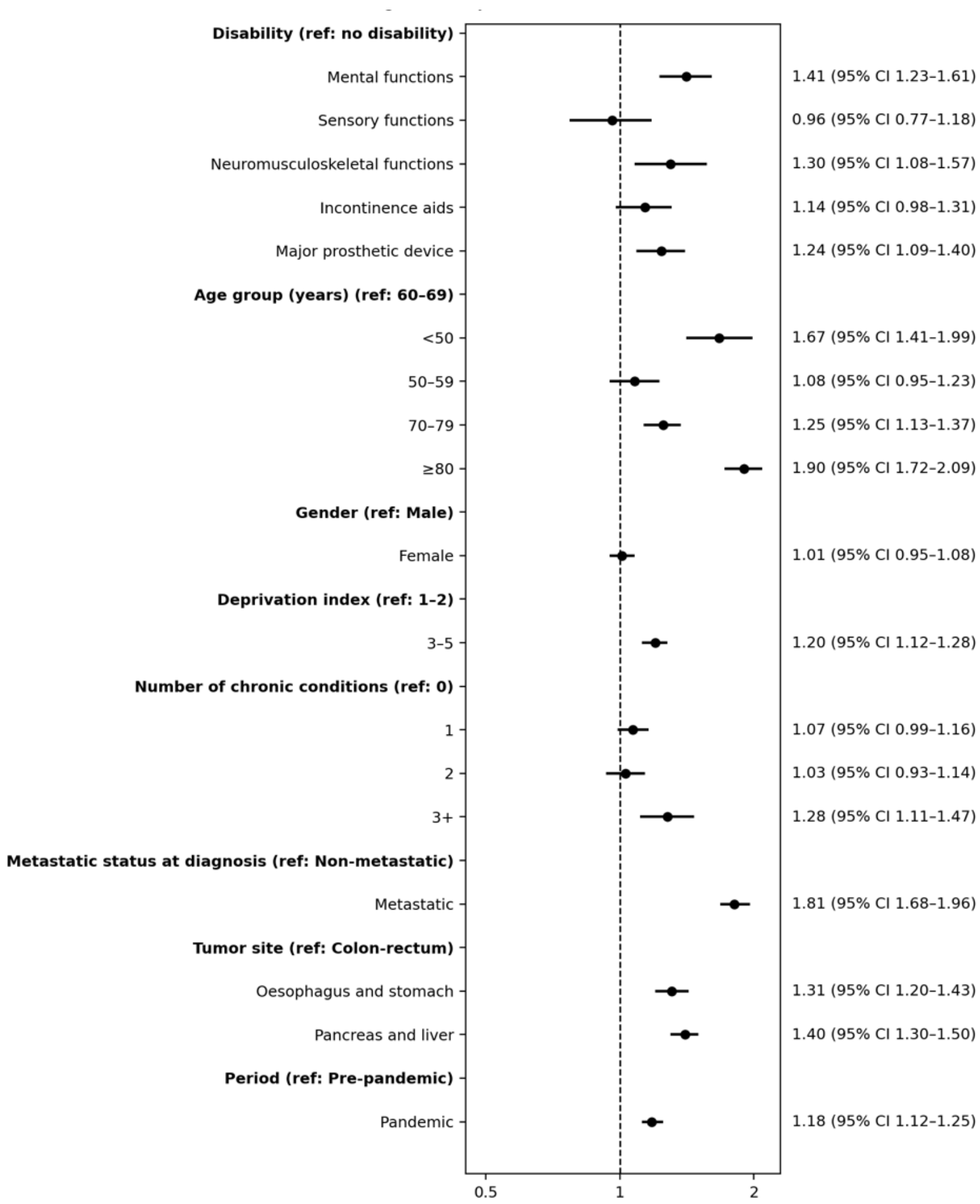

Legend: Multivariable logistic regression assessing factors associated with emergency presentation. Multivariable logistic regression assessing the association between cancer diagnosis following emergency presentation (EP) and ICF-based impairment categories (mental, sensory, neuromusculoskeletal and movement-related, continence-related, and prosthetic or orthotic-related), sociodemographic characteristics, number of comorbidities, metastatic status, period of diagnosis (pre-COVID-19 vs. COVID-19), and cancer site among patients with colorectal, upper gastrointestinal, and hepatopancreatobiliary cancers diagnosed in 2018–2021 in the provinces of Milan and Lodi, northern Italy (n=16,964). Estimates are derived from a multivariable logistic regression model. Adjusted odds ratios are reported with 95% confidence intervals. Wald tests for adjusted odds ratios are two-sided. No adjustment was made for multiple comparisons. Abbreviations: EP, emergency presentation; Ref, reference.

Supplementary Figure 2.

Colorectal cancer

a)

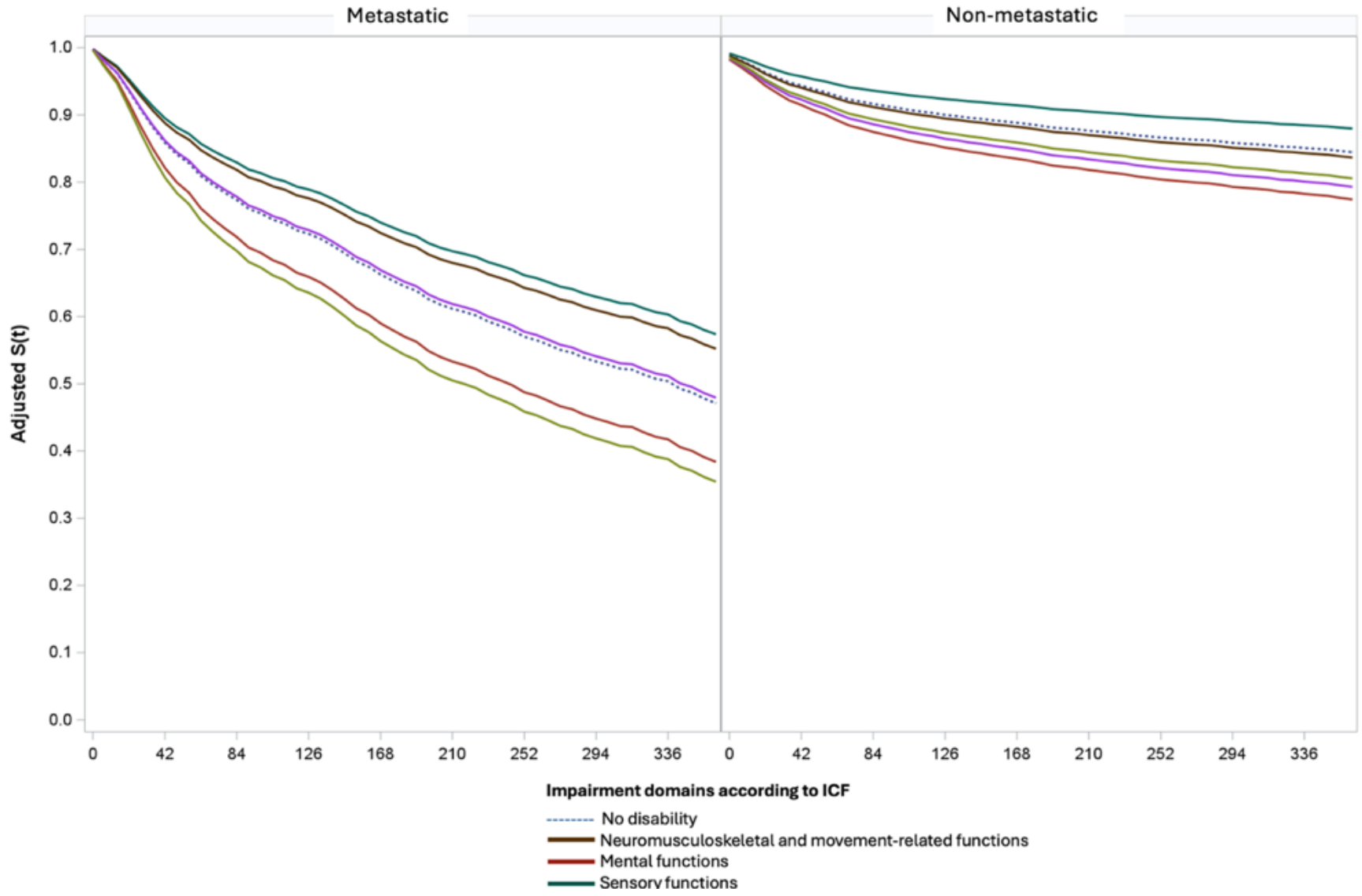

Hepatobiliary cancers

b)

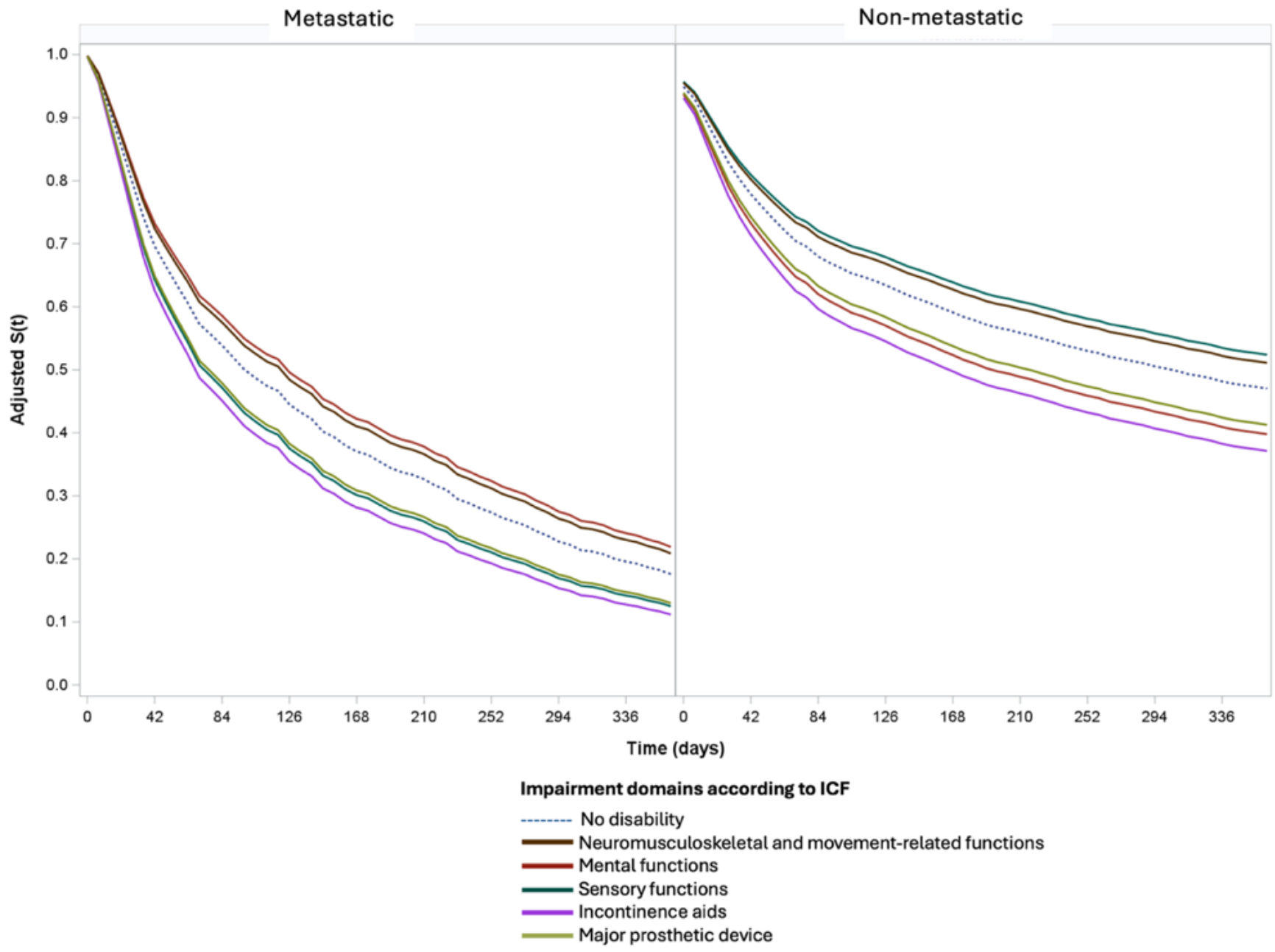

Upper gastrointestinal cancers

c)

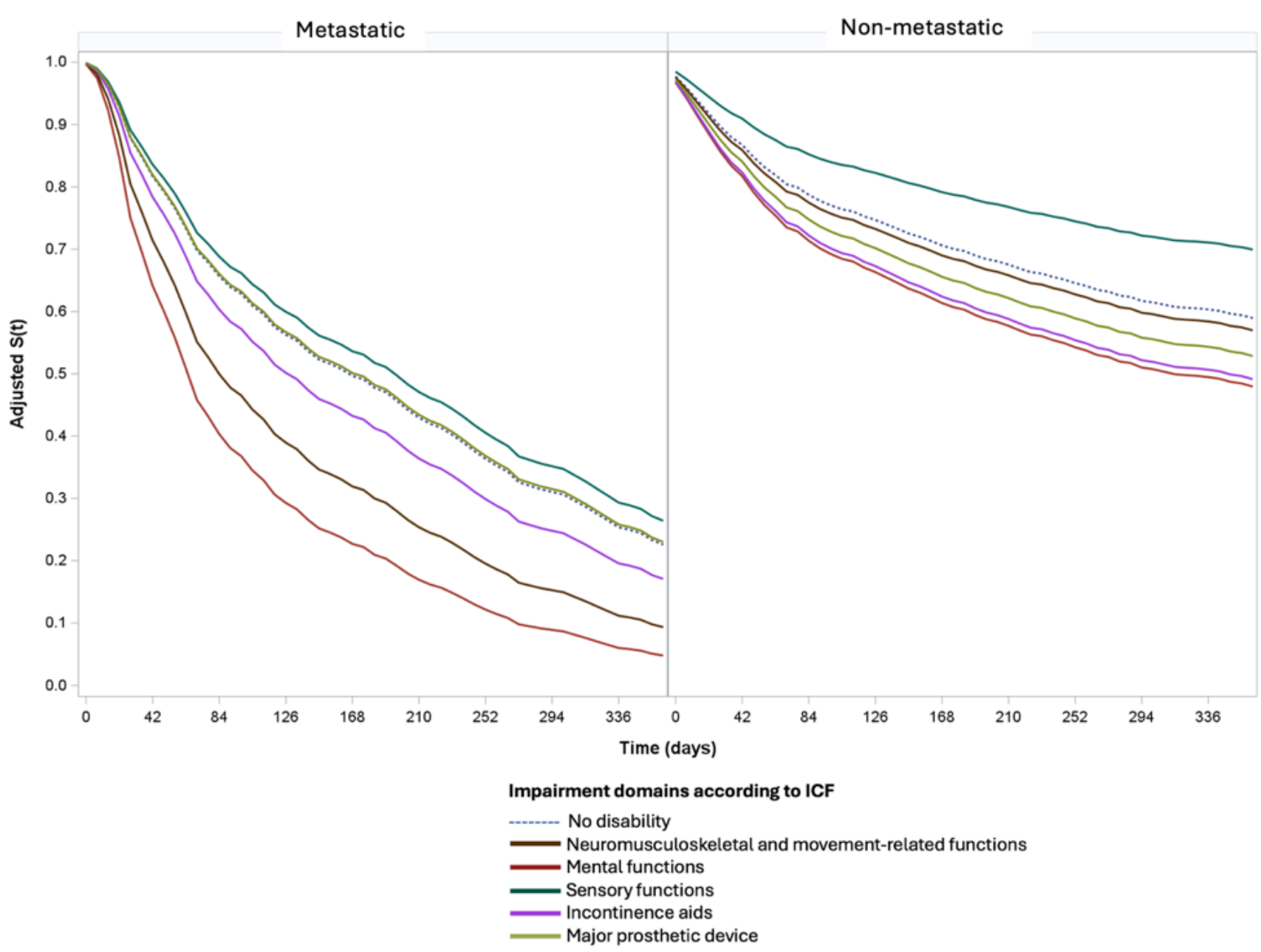

Legend: Adjusted one-year survival curves by metastatic status and tumour site.

Adjusted one-year survival curves are shown by metastatic status at diagnosis (metastatic vs. non-metastatic) and stratified by ICF-based impairment categories (mental functions, sensory functions, neuromusculoskeletal and movement-related functions, continence functions, and major prosthetic device use), compared with patients without impairments. Curves are shown separately by tumour site: (a) colorectal cancer (n=8,589), (b) hepatopancreatobiliary cancer (n=5,364), and (c) upper gastrointestinal cancer (n=3,011). All curves are derived from Cox proportional hazards models and adjusted for sex, age group, number of chronic conditions, diagnostic route (emergency presentation vs. screening/inpatient/outpatient visits), and period of diagnosis (pre-pandemic vs. pandemic), among incident cases diagnosed in 2018–2021 in the provinces of Milan and Lodi, northern Italy. No adjustment was made for multiple comparisons.

Abbreviations: ICF, International Classification of Functioning, Disability, and Health.

Supplementary Figure 3.

a)

Colorectal cancer

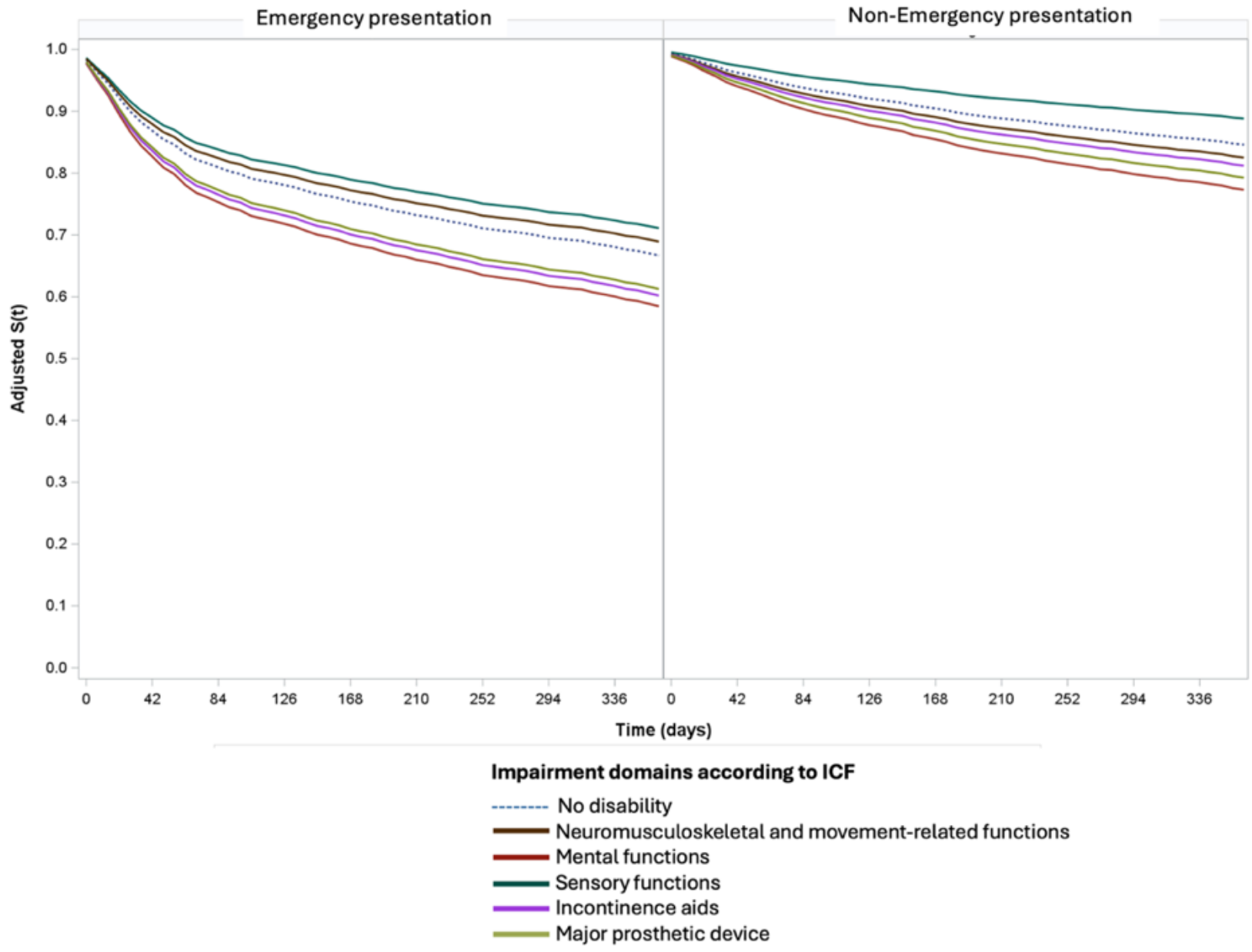

b)

Hepatobiliary cancers

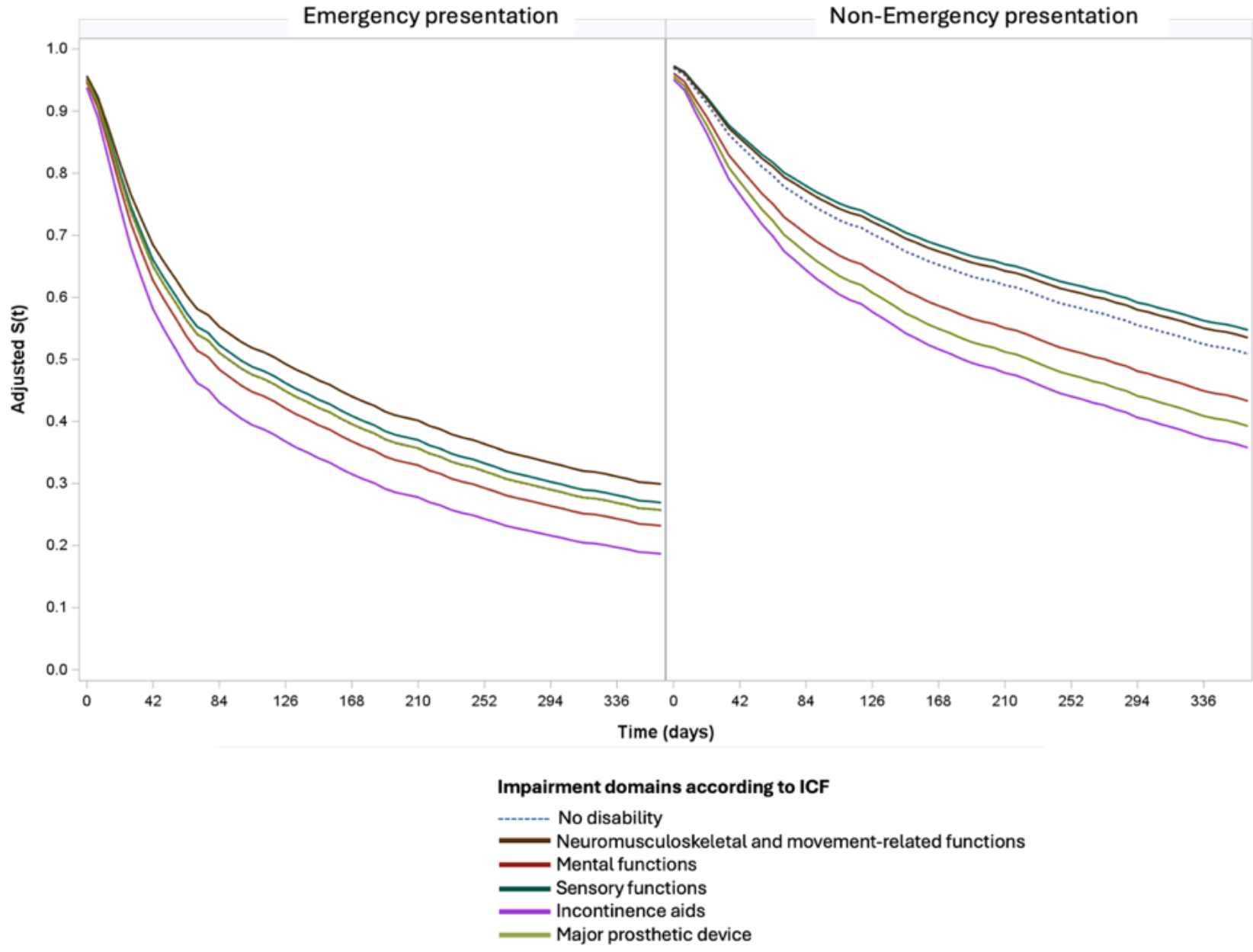

c)

Upper gastrointestinal cancers

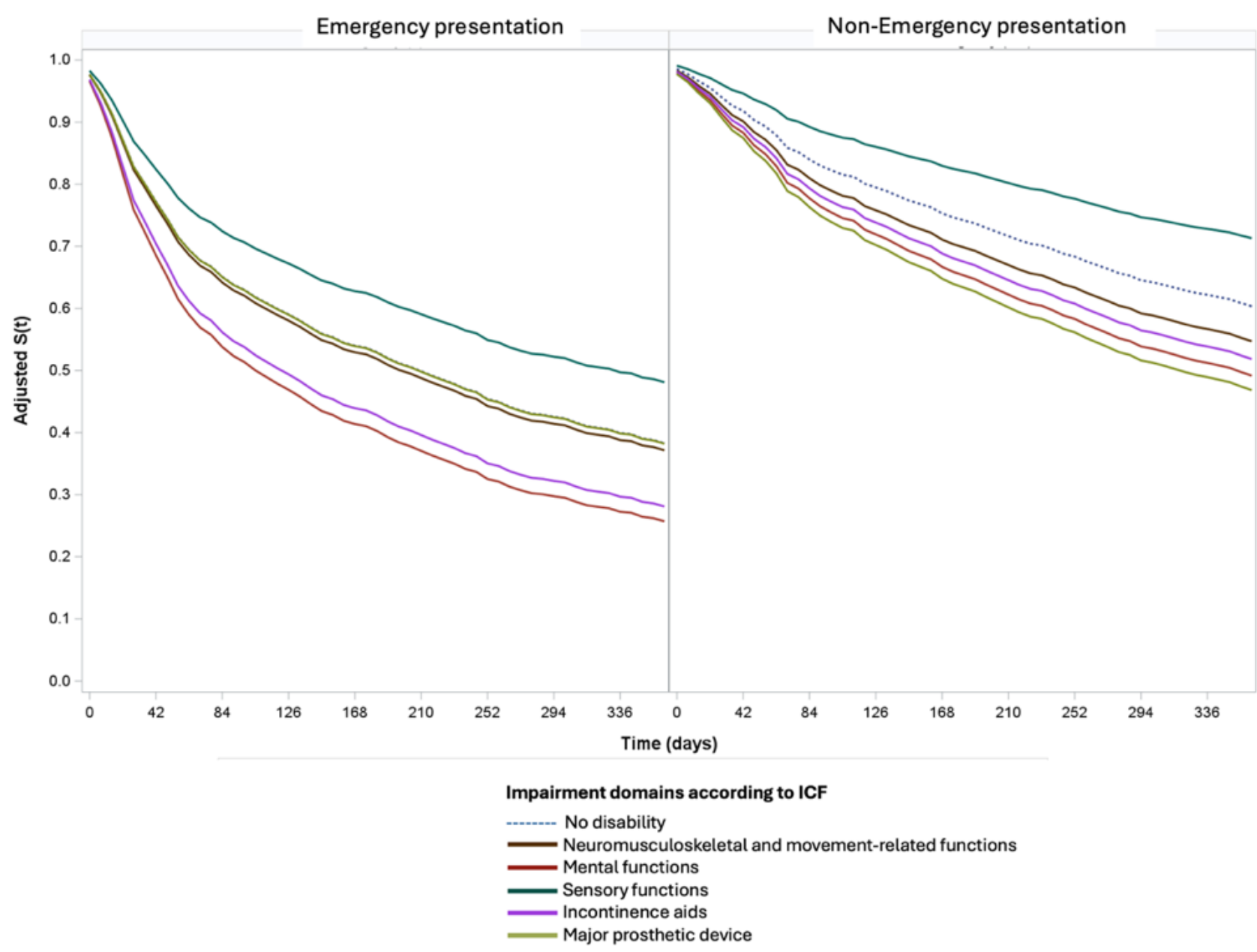

Legend: Adjusted one-year survival curves by diagnostic route and tumour site.

Adjusted one-year survival curves are shown by diagnostic route at diagnosis (emergency presentation vs. non-emergency: screening/inpatient/outpatient) and stratified by ICF-based impairment categories (mental functions, sensory functions and pain, neuromusculoskeletal and movement-related functions, continence functions, and prosthetic or orthotic use), compared with patients without impairments. Panels show results separately for (a) colorectal cancer (n=8,589), (b) hepatopancreatobiliary cancer (n=5,364), and (c) upper gastrointestinal cancer (n=3,011). Survival curves were estimated from Cox proportional hazards models adjusted for age group, sex, number of chronic conditions, metastatic status at diagnosis, and period (pre- vs. during COVID-19), among patients diagnosed in 2018–2021 in the provinces of Milan and Lodi, northern Italy. No adjustment was made for multiple comparisons.

Abbreviations: ICF, International Classification of Functioning, Disability, and Health.

Supplementary Table 1.

Diagnostic pathway	Colorectal cancer			Hepatopancreatobiliary cancer			Upper gastrointestinal cancer			All cancer		
	Disability		pvalue	Disability		pvalue	Disability		pvalue	Disability		pvalue
	Yes	No		Yes	No		Yes	No		Yes	No	
EP	751 (44.7)	2180 (32.2)	<0.001	474 (49.3)	1833 (42.4)	<0.001	283 (53.9%)	970 (39.4%)	<0.001	1508 (47.6%)	4983 (36.8%)	<0.001
IP/OP/screening	930 (55.3)	4597 (67.8)		487 (50.7)	2488 (57.6)		242 (46.1%)	1491 (60.6%)		1659 (52.4%)	8576 (63.3%)	

Legend: Diagnostic pathways by disability status and tumour site.

Diagnostic pathways are shown according to disability status and tumour site among patients with digestive system cancers diagnosed in 2018–2021 in the provinces of Milan and Lodi, northern Italy (n=16,964). Tumour site groups include colorectal cancer (n=8,589), hepatopancreatobiliary cancer (n=5,364), and upper gastrointestinal cancer (n=3,011). Overall, 3,222 patients had at least one pre-existing impairment and 13,742 had no documented pre-existing impairment. P values indicate differences in diagnostic pathway distributions between patients with and without pre-existing impairments and were calculated using Pearson's χ^2 test. All p values are two-sided. No adjustment was made for multiple comparisons.

Abbreviations: EP, emergency presentation; IP, inpatient; OP, outpatient.

Supplementary Table 2.

	Emergency presentation (EP)	
	**Pre- COVID-19 OR (95% CI)**	**During COVID-19 OR (95% CI)**
**Impairment categories** (ref: None)		
Impairments of mental functions	1.36 (1.13–1.64)	1.44 (1.19–1.75)
Impairments of sensory functions	1.13 (0.85–1.51)	0.79 (0.58–1.08)
Impairments of neuromusculoskeletal and movement-related functions	1.40 (1.07–1.83)	1.22 (0.94–1.60)
Impairments of continence functions	1.23 (1.02–1.49)	1.02 (0.82–1.28)
Impairments of functions related to prosthetic or orthotic use	1.16 (0.97–1.38)	1.32 (1.11–1.58)
**Cancer site** (ref: Colorectal)		
Hepatobiliary	1.36 (1.23–1.50)	1.44 (1.29–1.61)
Upper gastrointestinal	1.38 (1.22–1.56)	1.24 (1.09–1.41)
**Gender** (ref: Male)		
Female	1.00 (0.91–1.10)	1.03 (0.93–1.13)
**Age** (ref: 60–69)		
<50	1.94 (1.54–2.44)	1.40 (1.08–1.82)
50–59	1.10 (0.92–1.31)	1.06 (0.88–1.27)
70–79	1.31 (1.14–1.49)	1.18 (1.03–1.36)
≥80	1.92 (1.68–2.19)	1.88 (1.63–2.16)
**Deprivation index** (ref: Low)		
High deprivation	1.26 (1.15–1.39)	1.13 (1.02–1.24)
**Metastatic status** (ref: Non-metastatic)		
Metastatic disease	1.68 (1.52–1.87)	1.98 (1.76–2.23)
**Number of chronic conditions** (ref: None)		
1	1.13 (1.02–1.25)	1.01 (0.91–1.13)
2	1.05 (0.92–1.21)	1.02 (0.87–1.18)
≥3	1.35 (1.12–1.63)	1.22 (0.99–1.49)

Legend: Multivariable logistic regression assessing factors associated with emergency presentation by period of diagnosis. Multivariable logistic regression assessing the association between cancer site, sex, age group, deprivation index, number of chronic conditions, metastatic status, and impairment categories (mental functions, sensory functions and pain, neuromusculoskeletal and movement-related functions, continence functions, and prosthetic or orthotic use), with the risk of emergency presentation (EP) among patients with digestive system cancers diagnosed in the pre-COVID-19 (2018–2019) and COVID-19 (2020–2021) periods in the provinces of Milan and Lodi, northern Italy (n=16,964). Adjusted odds ratios are reported with 95% confidence intervals. Wald tests for adjusted odds ratios are two-sided. No adjustment was made for multiple comparisons.

Abbreviations: CI, confidence interval; EP, emergency presentation; Ref, reference.

Supplementary Table 3.

Metastatic status at diagnosis	Adjusted Hazard Ratio (95% CI)
**Non-metastatic**	
Impairments of mental functions	1.42 (1.30–1.54)
Impairments of sensory functions and pain	0.76 (0.66–0.87)
Impairments of neuromusculoskeletal and movement-related functions	0.99 (0.88–1.13)
Impairments of continence functions	1.39 (1.26–1.52)
Impairments related to prosthetic or orthotic use	1.26 (1.16–1.37)
**Metastatic**	
Impairments of mental functions	1.16 (0.98–1.38)
Impairments of sensory functions and pain	0.85 (0.63–1.14)
Impairments of neuromusculoskeletal and movement-related functions	0.95 (0.75–1.21)
Impairments of continence functions	1.07 (0.90–1.27)
Impairments related to prosthetic or orthotic use	1.27 (1.10–1.46)

Legend: Association between ICF-based impairment categories and one-year overall mortality, stratified by cancer metastatic status at diagnosis.

Associations between ICF-based impairment categories and one-year overall mortality are shown stratified by cancer metastatic status at diagnosis (non-metastatic vs. metastatic) among patients with digestive system cancers diagnosed in 2018–2021 in the provinces of Milan and Lodi, northern Italy (n=16,964). Estimates were obtained from multivariable Cox proportional hazards regression models adjusted for cancer site, sex, age group, deprivation index, number of chronic conditions, diagnostic route, and COVID-19 period. Hazard ratios are reported with 95% confidence intervals. Wald tests for hazard ratios are two-sided. No adjustment was made for multiple comparisons.

Abbreviations: CI, confidence interval; HR, hazard ratio; ICF, International Classification of Functioning, Disability, and Health.

Supplementary Table 4.

Diagnostic route	Adjusted Hazard Ratio (95% CI)
**Non-emergency presentation (EP = 0)**	
Impairments of mental functions	1.42 (1.27–1.59)
Impairments of sensory functions and pain	0.75 (0.63–0.90)
Impairments of neuromusculoskeletal and movement-related functions	1.08 (0.91–1.27)
Impairments of continence functions	1.39 (1.23–1.56)
Impairments related to prosthetic or orthotic use	1.50 (1.36–1.67)
**Emergency presentation (EP = 1)**	
Impairments of mental functions	1.28 (1.16–1.42)
Impairments of sensory functions and pain	0.84 (0.71–1.00)
Impairments of neuromusculoskeletal and movement-related functions	0.94 (0.81–1.09)
Impairments of continence functions	1.30 (1.15–1.45)
Impairments related to prosthetic or orthotic use	1.09 (0.99–1.20)

Legend: Association between ICF-based impairment categories and one-year overall mortality, stratified by diagnostic route.

Associations between ICF-based impairment categories and one-year overall mortality are shown stratified by diagnostic route (emergency vs. non-emergency presentation) among patients with digestive system cancers diagnosed in 2018–2021 in the provinces of Milan and Lodi, northern Italy (n=16,964). Estimates were obtained from multivariable Cox proportional hazards regression models adjusted for cancer site, sex, age group, deprivation index, number of chronic conditions, metastatic status, and COVID-19 period. Hazard ratios are reported with 95% confidence intervals. Wald tests for hazard ratios are two-sided. No adjustment was made for multiple comparisons.

Abbreviations: CI, confidence interval; EP, emergency presentation; HR, hazard ratio; ICF, International Classification of Functioning, Disability, and Health.

Supplementary Table 5.

Tumour site	Diagnostic pathway	Impairment domain	Hazard Ratio (95% CI)	p-value
Colorectal	Emergency	Mental functions	1.37 (1.17–1.61)	<0.001
		Incontinence aids	1.29 (1.09–1.51)	0.002
		Major prosthetic device	1.24 (1.06–1.44)	0.007
		Neuromusculoskeletal functions	0.91 (0.72–1.14)	0.419
		Sensory functions and pain	0.83 (0.63–1.08)	0.169
	Non-emergency	Mental functions	1.63 (1.37–1.93)	<0.001
		Incontinence aids	1.28 (1.08–1.51)	0.003
		Major prosthetic device	1.45 (1.22–1.71)	<0.001
		Neuromusculoskeletal functions	1.17 (0.91–1.50)	0.229
		Sensory functions and pain	0.69 (0.52–0.90)	0.007
Hepatopancreatobiliary	Emergency	Incontinence aids	1.27 (1.04–1.57)	0.021
		Mental functions	1.09 (0.91–1.29)	0.351
		Major prosthetic device	1.00 (0.85–1.17)	0.991
		Neuromusculoskeletal functions	0.88 (0.69–1.12)	0.281
		Sensory functions and pain	0.96 (0.72–1.28)	0.784
	Non-emergency	Incontinence aids	1.62 (1.31–1.99)	<0.001
		Major prosthetic device	1.45 (1.23–1.70)	<0.001
		Mental functions	1.28 (1.06–1.54)	0.011
		Neuromusculoskeletal functions	0.92 (0.69–1.22)	0.551
		Sensory functions and pain	0.88 (0.66–1.17)	0.386
Upper gastrointestinal	Emergency	Mental functions	1.48 (1.18–1.86)	<0.001
		Incontinence aids	1.37 (1.05–1.79)	0.021
		Major prosthetic device	1.00 (0.80–1.25)	0.983
		Neuromusculoskeletal functions	1.04 (0.72–1.48)	0.850
		Sensory functions and pain	0.74 (0.51–1.07)	0.106
	Non-emergency	Mental functions	1.47 (1.11–1.96)	0.008
		Major prosthetic device	1.59 (1.25–2.03)	<0.001
		Incontinence aids	1.35 (0.99–1.83)	0.056
		Neuromusculoskeletal functions	1.22 (0.87–1.72)	0.254
		Sensory functions and pain	0.64 (0.42–0.99)	0.044

Legend: Adjusted hazard ratios for one-year overall survival by tumour site and diagnostic pathway.

Adjusted hazard ratios for one-year overall survival are shown by tumour site and diagnostic pathway among patients with colorectal cancer (n=8,589), hepatopancreatobiliary cancer (n=5,364), and upper gastrointestinal cancer (n=3,011) diagnosed in 2018–2021 in the provinces of Milan and Lodi, northern Italy. Estimates were obtained from multivariable Cox proportional hazards regression models. Hazard ratios are reported with 95% confidence intervals. P values indicate the statistical significance of the corresponding hazard ratio estimates and were calculated using two-sided Wald tests. No adjustment was made for multiple comparisons.

Abbreviations: CI, confidence interval; HR, hazard ratio.

Supplementary Table 6.

Tumour site	Metastatic status	Impairment domain	Hazard Ratio (95% CI)	p-value
Colorectal	Non-metastatic	Mental functions	1.63 (1.37–1.93)	<0.001
		Incontinence aids	1.28 (1.08–1.51)	0.003
		Major prosthetic device	1.45 (1.22–1.71)	<0.001
		Neuromusculoskeletal functions	1.17 (0.91–1.50)	0.229
		Sensory functions and pain	0.69 (0.52–0.90)	0.007
	Metastatic	Mental functions	1.37 (1.17–1.61)	<0.001
		Incontinence aids	1.29 (1.09–1.51)	0.002
		Major prosthetic device	1.24 (1.06–1.44)	0.007
		Neuromusculoskeletal functions	0.91 (0.72–1.14)	0.419
		Sensory functions and pain	0.83 (0.63–1.08)	0.169
Hepatopancreatobiliary	Non-metastatic	Incontinence aids	1.62 (1.31–1.99)	<0.001
		Major prosthetic device	1.45 (1.23–1.70)	<0.001
		Mental functions	1.28 (1.06–1.54)	0.011
		Neuromusculoskeletal functions	0.92 (0.69–1.22)	0.551
		Sensory functions and pain	0.88 (0.66–1.17)	0.386
	Metastatic	Incontinence aids	1.27 (1.04–1.57)	0.021
		Mental functions	1.09 (0.91–1.29)	0.351
		Major prosthetic device	1.00 (0.85–1.17)	0.991
		Neuromusculoskeletal functions	0.88 (0.69–1.12)	0.281
		Sensory functions and pain	0.96 (0.72–1.28)	0.784
Upper gastrointestinal	Non-metastatic	Mental functions	1.47 (1.11–1.96)	0.008
		Major prosthetic device	1.59 (1.25–2.03)	<0.001
		Incontinence aids	1.35 (0.99–1.83)	0.056
		Neuromusculoskeletal functions	1.22 (0.87–1.72)	0.254
		Sensory functions and pain	0.64 (0.42–0.99)	0.044
	Metastatic	Mental functions	1.48 (1.18–1.86)	<0.001
		Incontinence aids	1.37 (1.05–1.79)	0.021
		Major prosthetic device	1.00 (0.80–1.25)	0.983
		Neuromusculoskeletal functions	1.04 (0.72–1.48)	0.850
		Sensory functions and pain	0.74 (0.51–1.07)	0.106

Legend: Adjusted hazard ratios for one-year overall survival by tumour site and metastatic status.

Adjusted hazard ratios for one-year overall survival are shown by tumour site and metastatic status among patients with colorectal cancer (n=8,589), hepatopancreatobiliary cancer (n=5,364), and upper gastrointestinal cancer (n=3,011) diagnosed in 2018–2021 in the provinces of Milan and Lodi, northern Italy. Estimates were obtained from multivariable Cox proportional hazards regression models. Hazard ratios are reported with 95% confidence intervals. P values indicate the statistical significance of the corresponding hazard ratio estimates and were calculated using two-sided Wald tests. No adjustment was made for multiple comparisons.

Abbreviations: CI, confidence interval; HR, hazard ratio.
