# Supplementary material for: Disability related disparities in diagnosis and survival of digestive system cancers in Northern Italy
Source: Nat Commun. 2026 Oct 10;17:11553. doi: 10.1038/s41467-026-78262-w (PMC13748588; doi:10.1038/s41467-026-78262-w)
Supplement: Supplementary file 2 [file 41467_2026_78262_MOESM2_ESM.pdf]

Description of Additional Supplementary Files

Supplementary Dataset 1. Characteristics of cancer patients with and without pre-existing impairments. Socio-demographic and clinical characteristics are shown for cancer patients with digestive system cancers diagnosed in 2018–2021 in the provinces of Milan and Lodi, northern Italy (n=16,964). Overall, 3,222 patients had at least one pre-existing impairment and 13,742 had no documented pre-existing impairment. P values indicate differences in characteristics between patients with and without pre-existing impairments and were calculated using Pearson's χ^2 test. All p values are two-sided. No adjustment was made for multiple comparisons.

Supplementary Dataset 2. Sequentially adjusted logistic regression models for the association between impairment categories and emergency presentation.

Odds ratios and 95% confidence intervals are shown for emergency presentation according to impairment categories and covariates among patients with digestive system cancers diagnosed in 2018–2021 in the provinces of Milan and Lodi, northern Italy (n=16,964). Model 1 was adjusted for age and sex; Model 2 was additionally adjusted for deprivation index and number of chronic conditions; Model 3 was further adjusted for cancer-related characteristics, including tumour site, metastatic status at diagnosis, and period of diagnosis (pre-pandemic vs. pandemic). P values indicate the statistical significance of the corresponding odds ratio estimates and were calculated using two-sided Wald tests. No adjustment was made for multiple comparisons.

Abbreviations: CI, confidence interval; EP, emergency presentation; OR, odds ratio; Ref, reference.
