# Supplementary material for: Disability related disparities in diagnosis and survival of digestive system cancers in Northern Italy
Source: Nat Commun. 2026 Oct 10;17:11553. doi: 10.1038/s41467-026-78262-w (PMC13748588; doi:10.1038/s41467-026-78262-w)
Supplement: Supplementary file 3 [file 41467_2026_78262_MOESM5_ESM.pdf]

nature portfolio

Corresponding author(s): Carlotta Buzzoni

Last updated by author(s): Apr 2, 2026

Reporting Summary

Nature Portfolio wishes to improve the reproducibility of the work that we publish. This form provides structure for consistency and transparency in reporting. For further information on Nature Portfolio policies, see our Editorial Policies and the Editorial Policy Checklist.

Statistics

For all statistical analyses, confirm that the following items are present in the figure legend, table legend, main text, or Methods section.

n/a	Confirmed	
☐	☒	The exact sample size (*n*) for each experimental group/condition, given as a discrete number and unit of measurement
☐	☒	A statement on whether measurements were taken from distinct samples or whether the same sample was measured repeatedly
☐	☒	The statistical test(s) used AND whether they are one- or two-sided<br>*Only common tests should be described solely by name; describe more complex techniques in the Methods section.*
☐	☒	A description of all covariates tested
☐	☒	A description of any assumptions or corrections, such as tests of normality and adjustment for multiple comparisons
☐	☒	A full description of the statistical parameters including central tendency (e.g. means) or other basic estimates (e.g. regression coefficient) AND variation (e.g. standard deviation) or associated estimates of uncertainty (e.g. confidence intervals)
☐	☒	For null hypothesis testing, the test statistic (e.g. *F*, *t*, *r*) with confidence intervals, effect sizes, degrees of freedom and *P* value noted<br>*Give P values as exact values whenever suitable.*
☒	☐	For Bayesian analysis, information on the choice of priors and Markov chain Monte Carlo settings
☐	☒	For hierarchical and complex designs, identification of the appropriate level for tests and full reporting of outcomes
☐	☒	Estimates of effect sizes (e.g. Cohen's *d*, Pearson's *r*), indicating how they were calculated

Our web collection on statistics for biologists contains articles on many of the points above.

Software and code

Policy information about availability of computer code

Data collection	version 9.4; SAS Institute Inc., Cary, NC, USA
Data analysis	version 9.4; SAS Institute Inc., Cary, NC, USA

For manuscripts utilizing custom algorithms or software that are central to the research but not yet described in published literature, software must be made available to editors and reviewers. We strongly encourage code deposition in a community repository (e.g. GitHub). See the Nature Portfolio guidelines for submitting code & software for further information.

Data

Policy information about availability of data

All manuscripts must include a data availability statement. This statement should provide the following information, where applicable:

- Accession codes, unique identifiers, or web links for publicly available datasets
- A description of any restrictions on data availability
- For clinical datasets or third party data, please ensure that the statement adheres to our policy

The data supporting the findings of this article are available at an aggregated level from the authors upon reasonable request and with permission of ATS of Milan. Requests to access should be directed to Carlotta Buzzoni, Scientific Manager of the study, email: cbuzzoni@ats-milano.it

Research involving human participants, their data, or biological material

Policy information about studies with human participants or human data. See also policy information about sex, gender (identity/presentation), and sexual orientation and race, ethnicity and racism.

Reporting on sex and gender	Sex (biological attribute) was obtained from cancer registry data and included as an a priori covariate in all statistical models. Descriptive statistics were reported separately for males and females. Gender-related variables were not available in the data sources and could not be assessed.
Reporting on race, ethnicity, or other socially relevant groupings	Race and ethnicity data were not collected in the administrative and cancer registry sources used for this study. As a result, analyses were conducted using available socio-demographic variables, including age, sex, and area-level deprivation, to account for potential social inequalities.
Population characteristics	Population characteristics were described using socio-demographic and clinical variables, including age, sex, area-level socioeconomic deprivation, cancer site, metastatic status at diagnosis, number of chronic conditions, and period of diagnosis (pre- and during COVID-19).
Recruitment	No active recruitment was performed. The study included all eligible cases identified through population-based cancer registry data linked with administrative healthcare databases.
Ethics oversight	The study was approved by the Ethics Committee "Comitato Etico Territoriale Lombardia 3" (approval number 5328_20.11.2024_P). All data were anonymised prior to analysis in accordance with applicable regulations.

Note that full information on the approval of the study protocol must also be provided in the manuscript.

Field-specific reporting

Please select the one below that is the best fit for your research. If you are not sure, read the appropriate sections before making your selection.

☐ Life sciences ☒ Behavioural & social sciences ☐ Ecological, evolutionary & environmental sciences

For a reference copy of the document with all sections, see nature.com/documents/nr-reporting-summary-flat.pdf

Behavioural & social sciences study design

All studies must disclose on these points even when the disclosure is negative.

Study description	This study is a retrospective population-based cohort study based on linked cancer registry and administrative healthcare data, aimed at examining disparities in diagnostic routes and one-year survival among patients with digestive system cancers according to pre-existing impairment status.
Research sample	The research sample included all individuals diagnosed with upper gastrointestinal, colorectal, and hepatopancreatobiliary cancers between 2018 and 2021 in the provinces of Milan and Lodi. This population-based sample is representative of the underlying resident population, as it is derived from a high-quality cancer registry covering a well-defined geographic area and linked to routinely collected administrative healthcare data. The use of comprehensive registry data ensures near-complete case ascertainment, minimising selection bias.<br><br>The study is based on linked datasets from the Milan Cancer Registry and administrative healthcare databases of the Agency for Health Protection (ATS) of Milan. These data sources include information on cancer diagnosis, tumour characteristics, and patient demographics, as well as healthcare utilisation records such as hospital admissions, outpatient visits, and social and healthcare service data. Deterministic linkage was performed using anonymised individual identifiers, enabling the integration of clinical and healthcare pathway information at the individual level.
Sampling strategy	No sampling strategy was applied, as the study is population-based and includes all eligible cancer cases identified through the cancer registry. This approach ensures comprehensive coverage of the target population and reduces the risk of selection bias.
Data collection	Data were collected from routinely recorded population-based cancer registry data and linked administrative healthcare and social care databases. Deterministic linkage using anonymised individual identifiers enabled the integration of information on cancer characteristics, socio-demographic factors, healthcare utilisation, and pre-existing impairments at the individual level.
Timing	The study included cancers diagnosed between 2018 and 2021. Data on healthcare utilisation were collected for the 6 months preceding cancer diagnosis to define diagnostic routes, and patients were followed up for one year after diagnosis to assess survival.
Data exclusions	No additional data exclusions were applied beyond eligibility criteria; however, impairment categories with low prevalence (<2%) were excluded from analyses to ensure robustness of estimates.
Non-participation	Non-participation is not applicable, as the study is based on routinely collected population-based data and includes all eligible cases identified through registry linkage.

Randomization	Randomization was not performed, as this was an observational retrospective population-based cohort study using routinely collected data.

Reporting for specific materials, systems and methods

We require information from authors about some types of materials, experimental systems and methods used in many studies. Here, indicate whether each material, system or method listed is relevant to your study. If you are not sure if a list item applies to your research, read the appropriate section before selecting a response.

Materials & experimental systems

n/a	Involved in the study
☒	☐ Antibodies
☒	☐ Eukaryotic cell lines
☒	☐ Palaeontology and archaeology
☒	☐ Animals and other organisms
☒	☐ Clinical data
☒	☐ Dual use research of concern
☒	☐ Plants

Methods

n/a	Involved in the study
☒	☐ ChIP-seq
☒	☐ Flow cytometry
☒	☐ MRI-based neuroimaging

Plants

Seed stocks	na
Novel plant genotypes	na
Authentication	na
