# Supplementary material for: Disability related disparities in diagnosis and survival of digestive system cancers in Northern Italy
Source: Nat Commun. 2026 Oct 10;17:11553. doi: 10.1038/s41467-026-78262-w (PMC13748588; doi:10.1038/s41467-026-78262-w)
Supplement: Supplementary file 4 [file 41467_2026_78262_MOESM6_ESM.pdf]

nature portfolio

# Peer Review File

# Disability related disparities in diagnosis and survival of digestive system cancers in Northern Italy

Corresponding Author: Dr Carlotta Buzzoni

**This file contains all reviewer reports in order by version, followed by all author rebuttals in order by version.**

Version 0:

Reviewer comments:

Reviewer #1

(Remarks to the Author)
This paper explores disability-related disparities in the diagnosis of and survival from digestive system cancers using electronic health data from Italy. This topic is important and under-explored, and the paper has the potential to add value to the literature. However, there are some limitations of the paper in its current format.

1. Some digestive system cancers (e.g. colon) can be screened for, while others cannot (e.g. pancreas). It would therefore be helpful to look at different types of cancers more distinctly. For instance, disparities in how cancers are diagnosed for colon cancer may indicate low uptake of screening among disabled people, whereas for pancreatic cancer it may indicate difficulties accessing healthcare to obtain early diagnosis. Moreover, the authors have not provided sufficient information on the screening programmes in Italy, or their uptake, to allow the author to consider this aspect.

2. A second issue is that I have some concerns about the determination of disability. Disability determination based on diagnosis of specific conditions has limitations, as it does not take account of severity. The groupings are also not consistent with most approaches to describing disability type – psychosocial, sensory (hearing and vision), physical and intellectual/cognitive. Moreover, impairments of continence is not usually included in disability determination and may be a sign of the cancer (e.g. colon cancer). At a minimum, I would discard the impairments of continence category.

3. I have some concern with how the data are analysed. Disability is strongly related to age, and so crude analyses will likely be confounded by age and should be avoided. At a minimum, analyses should be age (and potentially sex) adjusted. On the other hand, the adjusted odds ratios are heavily adjusted, which may also adjust for variables on the causal pathway. Potentially the authors could adjust more sequentially for additional variables.

4. The authors have not provided a clear rationale as to why there may be disparities in diagnosis and survival, which would explain their analysis approach (e.g. indicators included in multivariable analysis).

5. The analyses related to differences in survival by disability status are not presented very clearly in my view, and are currently overly complex. I suggest presenting these separately for different cancer types, as survival is so variable, and then adjusting for factors such as stage at diagnosis.

Minor points
- I suggest presenting variables (including p values) to 2 decimal places.
- Table 1 is very large and should be separated – perhaps presenting baseline characteristics separate to the main outcomes of diagnostic and metastatic status.
- I believe that valuable information is lost by presenting figures rather than tables and numbers, so suggest that the figures are moved to the appendix and the tables are used instead.
- The discussion is somewhat long, and includes speculation in some sections.

Reviewer #2

(Remarks to the Author)
Majors
1. The manuscript addresses an important topic but the conceptual novelty relative to existing literature remains somewhat limited. The authors should clarify what new scientific or clinical insights this study provides beyond previous epidemiological studies on cancer inequalities.
2. The analyses demonstrate associations between impairment categories and emergency presentation; however, the mechanisms underlying these disparities remain largely speculative. The discussion proposes explanations such as diagnostic overshadowing or barriers to healthcare access, but these mechanisms are not directly evaluated in the analysis. The authors should acknowledge this limitation and avoid overinterpretation of causal pathways.
3. Disability status is derived from administrative indicators mapped to ICF categories. This approach may not fully capture the severity or functional impact of impairments and could introduce misclassification bias. So, additional justification, validation references, or sensitivity analyses are needed for supporting this classification approach.
4. Although the models adjust for several relevant variables, important potential confounders remain unmeasured, such as health behaviours, healthcare utilization patterns, frailty, and health literacy. These factors could influence both impairment status and the likelihood of emergency diagnosis, potentially affecting the estimated associations.
5. The observation that sensory impairments are associated with lower mortality in some analyses is interesting but insufficiently explained. The authors should provide a more detailed interpretation of this finding and discuss possible explanations such as detection bias, healthcare utilization patterns, or residual confounding.
6. While the manuscript highlights disparities in cancer diagnosis among individuals with impairments, the discussion could more clearly articulate the clinical and health system implications of these findings. More specific recommendations regarding screening pathways, diagnostic processes, or healthcare accessibility for disabled populations would strengthen the translational relevance of the study.
Minors
1. The rationale for focusing on one-year overall survival should be clarified, particularly given the varying survival trajectories across the cancer types included in the analysis.
2. Several stratified analyses are presented (e.g., by metastatic status and diagnostic route), but the clinical interpretation of these results could be more clearly explained in the text.
3. The potential impact of COVID-19 on diagnostic pathways and healthcare access is only briefly discussed. Additional contextualization would improve interpretation.
4. The adjusted survival curves presented in the supplementary are somewhat complex. Clearer labeling or highlighting of key differences between impairment groups would improve readability.

Version 1:

Reviewer comments:

Reviewer #1

(Remarks to the Author)
The authors have responded well to my comments.

Reviewer #2

(Remarks to the Author)
The authors have substantially improved the manuscript and have addressed the concerns raised in the previous review. I recommend acceptance.

**Open Access** This Peer Review File is licensed under a Creative Commons Attribution 4.0 International License, which permits use, sharing, adaptation, distribution and reproduction in any medium or format, as long as you give appropriate credit to the original author(s) and the source, provide a link to the Creative Commons license, and indicate if changes were made.
In cases where reviewers are anonymous, credit should be given to 'Anonymous Referee' and the source.
The images or other third party material in this Peer Review File are included in the article's Creative Commons license, unless indicated otherwise in a credit line to the material. If material is not included in the article's Creative Commons license and your intended use is not permitted by statutory regulation or exceeds the permitted use, you will need to obtain permission directly from the copyright holder.
To view a copy of this license, visit https://creativecommons.org/licenses/by/4.0/

# Reviewer 1

| | |
|---|---|
| This paper explores disability-related disparities in the diagnosis of and survival from digestive system cancers using electronic health data from Italy. This topic is important and under-explored, and the paper has the potential to add value to the literature. However, there are some limitations of the paper in its current format | We thank the reviewer for this positive and encouraging comment. We appreciate the recognition of the relevance and potential contribution of our study to an important and under-explored area. We have carefully considered the points raised and have revised the manuscript accordingly to address the identified limitations and strengthen the overall clarity and methodological robustness of the paper. |
| 1. Some digestive system cancers (e.g. colon) can be screened for, while others cannot (e.g. pancreas). It would therefore be helpful to look at different types of cancers more distinctly. For instance, disparities in how cancers are diagnosed for colon cancer may indicate low uptake of screening among disabled people, whereas for pancreatic cancer it may indicate difficulties accessing healthcare to obtain early diagnosis. Moreover, the authors have not provided sufficient information on the screening programmes in Italy, or their uptake, to allow the author to consider this aspect. | We thank the reviewer for this thoughtful and helpful comment. We agree that the interpretation of diagnostic pathways may differ across cancer sites depending on whether organised screening programmes are available. In line with this comment, we have examined diagnostic pathways and survival both overall and stratified by tumour site (colorectal, hepatopancreatobiliary, and upper gastrointestinal cancers). We have now included a supplementary table (Table S1) presenting diagnostic pathways by disability status, stratified by tumour site. We have also reported these findings in the main text: "EP was consistently more frequent among patients with disabilities across all cancer types: 44.7% vs 32.2% in colorectal cancer, 49.3% vs 42.4% in HPB cancer, and 53.9% vs 39.4% in upper gastrointestinal cancer. Overall, this pattern persisted when considering all cancers combined (47.6% vs 36.8%) (Table S1).". Moreover, we have included Supplementary Figures S1 and S2, showing adjusted one-year survival curves by metastatic status, diagnostic route and stratified by tumor site.<br><br>In the Results section of the main manuscript, we are showing the overall estimate of the association between disability status and emergency presentation, adjusting for tumour site, considering that the association was consistent across tumour sites, with no evidence of effect modification. This has now been complemented with the presentation of the stratified analyses both in the Supplementary material and in the text of the main manuscript. However, if the Editors consider it more appropriate, we would be happy to include the stratified results by tumour site (currently presented in Table S1) in the main manuscript.<br><br>To further address the reviewer's comments, we have expanded the Introduction to describe the organisation of cancer screening programmes in |

| | |
|---|---|
| | Italy and in the Lombardy region. In particular, organised population-based colorectal cancer screening programmes in Lombardy are based on biennial faecal immunochemical testing (FIT), with invitations offered to individuals aged 50–74 years. We have also added information on screening uptake, noting that participation remains suboptimal (approximately 42–45% in recent years), which may contribute to inequalities in diagnostic pathways. No organised screening programmes are currently available for the other digestive system cancers included in this study.<br><br>We have also expanded the Discussion to clarify how differences in screening availability across cancer types may influence the interpretation of diagnostic pathways. In particular, for cancers without organised screening programmes disparities in EP are likely reflecting barriers in accessing timely diagnostic assessment among symptomatic individuals, whereas disparities observed for colorectal cancer may also reflect differences in access to screening among people with disabilities. Notably, even for cancers where screening is available, such as colorectal cancer, most cases are still diagnosed following symptomatic presentation rather than through screening programmes, supporting our focus on emergency presentation as a key indicator of inequalities in diagnostic pathways (Pennisi F et al, JAMA Netw Open. 2025). |
| 2. A second issue is that I have some concerns about the determination of disability. Disability determination based on diagnosis of specific conditions has limitations, as it does not take account of severity. The groupings are also not consistent with most approaches to describing disability type – psychosocial, sensory (hearing and vision), physical and intellectual/cognitive. Moreover, impairments of continence is not usually included in disability determination and may be a sign of the cancer (e.g. colon cancer). At a minimum, I would discard the impairments of continence category. | We thank the reviewer for this important comment. We agree that disability determination based on electronic health records and administrative data may not fully capture the severity or the full functional impact of impairments, and that some degree of misclassification is possible when functional status cannot be directly measured. We have now clarified this aspect more explicitly in the revised manuscript.<br><br>Our decision to use the International Classification of Functioning, Disability and Health (ICF), as the framework for defining impairment categories, was based on the recognition that health and disability cannot be fully understood based solely on diagnostic classifications. The ICF was specifically developed to complement and integrate traditional disease-based systems, such as ICD, by capturing the impact of health conditions on functioning (*Iezzoni LI. Using administrative data to study persons with disabilities. Milbank Q. 2002;80(2):347–379. doi: 10.1111/1468-* |

| | |
|---|---|
| | *0009.00023*). Compared with ICD-based approaches, the ICF provides a more function-oriented representation of health, allowing a more comprehensive characterisation of individuals who may share the same diagnosis, but differ substantially in their functional status.<br><br>While the ICF is considered an internationally recognised standard for describing functioning and disability (*Leonardi M et al. doi: 10.3390/ijerph191811321*), we acknowledge that the ICF categories do not directly correspond to frequently used disability typologies (e.g., psychosocial, sensory, physical, intellectual/cognitive). However, most studies on cancer inequalities typically focus on single conditions or selected disability groups (e.g., learning disability) and rely on diagnostic codes (e.g., SNOMED codes or ICD-based definitions), with a lack of studies examining multiple disability domains. In this context, the use of an ICF-based classification represents a novel approach and a key strength of our study, as it enables the simultaneous investigation of different impairment domains within a common conceptual framework. This is also consistent with previous evidence, supporting the validity of using routinely collected data for mapping ICF domains in the absence of direct functional assessments *(Maritz R et al, doi: 10.4338/ACI-2017050078;* Kukafka et al doi: 10.1197/jamia.M2107; Friedman DJ et al doi: 10.1111/jppi.12220).<br><br>We agree with the reviewer's comment that impairments of continence functions may, in some cases, reflect manifestations of advanced disease rather than a pre-existing disability. Even though in our study, continence-related impairments were recorded prior to cancer diagnosis, we acknowledge that some degree of misclassification cannot be excluded and we have now explicitly addressed this point in the Limitations section.<br><br>We also clarify that the continence domain encompasses both urinary and faecal incontinence, which may reflect underlying chronic conditions rather than cancer-related symptoms.<br><br>Importantly, all analyses were conducted separately for each impairment category; therefore, any potential misclassification related to continence impairments would not affect the associations observed for other impairment domains.  Moreover, this applies only to CRC cancer and not to other cancers, because incontinence is a symptom |

| | |
|---|---|
| | specifically associated with this type of tumor and is less commonly observed in the other cancers analyzed.<br><br>For these reasons, and to ensure consistency with the ICF-based framework and the validated algorithm used to identify impairments in administrative data, we retained this category in the analyses. However, we have clarified its rationale in the Methods section and expanded the Limitations section stating “In addition, for some impairment domains, such as continence functions, residual misclassification cannot be excluded, as these indicators may in some cases reflect manifestations of advanced disease rather than pre-existing disability, particularly in colorectal cancer. The impairment groupings used in this study reflect the ICF-based classification available in the administrative datasets and may not fully correspond to other commonly used disability typologies.”. |
| 3. I have some concern with how the data are analysed. Disability is strongly related to age, and so crude analyses will likely be confounded by age and should be avoided. At a minimum, analyses should be age (and potentially sex) adjusted. On the other hand, the adjusted odds ratios are heavily adjusted, which may also adjust for variables on the causal pathway. Potentially the authors could adjust more sequentially for additional variables. | We thank the reviewer for this important methodological comment.<br><br>We agree that age is strongly associated with disability. For this reason, all regression models presented in the manuscript were adjusted for age and sex. We have now clarified this more explicitly in the Methods section.<br><br>Regarding the reviewer’s concern about potential over-adjustment, the covariates included in the fully adjusted models were selected a priori based on previous evidence and their potential role as confounders of the association between impairment and cancer diagnostic pathways. These variables include sociodemographic characteristics and health-related factors that may be associated both with disability status and with access to diagnostic care (e.g. comorbidity burden…). We have specified this more clearly in the Methods section.<br><br>Moreover, in response to the reviewer’s suggestions we have now described in the Methods section that we have sequentially adjust for additional potential confounders employing three multivariable logistic regression models. These models and relevant findings are also reported in the Results-Table 2, where we present sequentially adjusted models. In particular, Model 1 adjusts for age and sex; Model 2 additionally includes deprivation and comorbidity burden; and Model 3 further adjusts for cancer-related factors, including cancer site, metastatic |

| | |
|---|---|
| | status and period of diagnosis. The Methods section has been revised to clarify this modelling strategy. In the Results (section 3.2 Multivariable analyses estimating the likelihood of EP by impairments and patient characteristics) we have also highlighted that the associations between disability and EP has remained very similar across the three models. |
| 4. The authors have not provided a clear rationale as to why there may be disparities in diagnosis and survival, which would explain their analysis approach (e.g. indicators included in multivariable analysis). | We agree that providing a clearer conceptual rationale for the disparities in cancer diagnosis and survival among individuals with disabilities can help to better contextualise the analytical approach. In response to the reviewer's comment, we have expanded both the Introduction and the Discussion to more clearly describe the mechanisms through which disability may influence diagnostic pathways and survival. In particular, individuals with disabilities may encounter multiple barriers across the cancer diagnostic pathway, including reduced access to screening, difficulties in recognising or communicating symptoms, physical or organisational barriers to accessing diagnostic testing, and the potential for diagnostic overshadowing in clinical settings. These factors may increase the likelihood of emergency presentation and, consequently, may adversely affect cancer prognosis and survival.<br><br>The Methods section has also been revised to clarify the rationale underlying the selection of covariates included in the multivariable models. In particular, we now clarify that the variables included in the multivariable models were selected a priori based on existing literature and on clinical and epidemiological reasoning, as well as considering the variables available within the linked electronic health databases used in this study. We have included sociodemographic characteristics (age, sex, and deprivation), comorbidity burden, and cancer-related factors (cancer site and metastatic status), all of which are known to influence access to care and cancer outcomes.<br><br>We acknowledge that some potentially relevant factors, such as symptom presentation, health literacy, and patterns of healthcare utilisation, are not available in administrative data and therefore could not be included in our models. We have explicitly acknowledged this limitation in the Discussion section. |
| 5. The analyses related to differences in survival by disability status are not presented very clearly in my view, and are currently overly complex. I suggest presenting these separately for different cancer types, as survival is so variable, and then adjusting for factors such as stage at diagnosis. | We agree that survival varies substantially across cancer types and that presenting results stratified by tumour site can improve interpretability.<br><br>In response, we have revised and streamlined the presentation of the survival analyses in the Results |

| | |
|---|---|
| | section (Section 3.3, “Overall survival stratified by impairment status, cancer metastatic status and diagnostic route”) to improve clarity and readability. We have also expanded the reporting of site-specific analyses. In particular, survival patterns stratified by tumour site are now presented in Supplementary Figures S1 and S2, where results are shown separately for colorectal, upper gastrointestinal, and hepatopancreatobiliary cancers. In addition, in response to the reviewer’s suggestion, cancer site–specific estimates are now additionally reported in the Results section and in the supplementary materials, including newly added Supplementary Tables S5 and S6 reporting hazard ratios stratified by tumour site.<br><br>In the main manuscript, we present the overall multivariable survival models, adjusted for cancer site, as the association between impairment status and reduced survival appeared broadly consistent across tumour sites. Formal testing for interaction between tumour site and impairment status did not show statistically significant interaction. Therefore, tumour site and metastatic status were included as covariates in the multivariable models. We believe this approach is useful for providing an overall estimate of the increased hazard of death associated with having a disability compared with no disability, independently of cancer type and other covariates, while also reducing the number of figures in the main manuscript and maintaining a clear and focused presentation.<br><br>If the Editor considers it preferable, we would be happy to include tumour site–specific survival curves in the main manuscript, rather than only showing them as supplementary material. |
| Minor points<br>- I suggest presenting variables (including p values) to 2 decimal places.<br>- Table 1 is very large and should be separated – perhaps presenting baseline characteristics separate to the main outcomes of diagnostic and metastatic status.<br>- I believe that valuable information is lost by presenting figures rather than tables and numbers, so suggest that the figures are moved to the appendix and the tables are used instead.<br>- The discussion is somewhat long, and includes speculation in some sections. | - We thank the reviewer for this suggestion. We have reported hazard ratios and odds ratios including two decimal places. Only when reporting percentage presented in Table 1, we have opted for one decimal place, as we believe this improves readability of the table. For p-values, we have reported three decimal places to ensure adequate precision. We will, of course, adapt the formatting as required by the journal guidelines at the production stage, if needed.<br><br>-We thank the reviewer for the helpful suggestion regarding Table 1. We have revised the table to improve clarity and readability. Specifically, we have reduced its size by removing variables that were not included in the subsequent multivariable analyses (i.e., educational level and marital status). |

| | |
|---|---|
| | As a result, the table is now more focused and easier to interpret.<br><br>- We have revised the presentation of the results in line with the suggestions, moving Figure 1 to the Supplementary Materials. We have included a new table (Table 2) in the main manuscript reporting the results of the sequentially adjusted logistic regression models. We believe that this improves the clarity and interpretability of the findings by providing detailed numerical estimates in the main text.<br><br>-We thank the reviewer for this helpful comment. In response, we have revised the Discussion section to improve clarity, and we have reduced or removed sections that were more speculative, focusing instead on interpretations more directly supported by our findings. |

## Reviewer 2

| | |
|---|---|
| 1. The manuscript addresses an important topic but the conceptual novelty relative to existing literature remains somewhat limited. The authors should clarify what new scientific or clinical insights this study provides beyond previous epidemiological studies on cancer inequalities. | We thank the reviewer for this important comment and for the opportunity to better clarify the scientific and clinical contribution of our study. In response, we have revised the Abstract, Introduction and Discussion to more explicitly highlight the novelty of the work and its contribution.<br><br>Specifically, this study contributes to the existing literature in several ways. First, to our knowledge, this is the first European population-based study to examine the risk of emergency cancer diagnosis, a route consistently associated with poorer prognosis, across multiple impairment categories within a unified analytical framework. Previous research on cancer disparities in people with disabilities has largely focused on individuals with intellectual or learning disabilities, with no population-based evidence available for other impairments. Our study adopts an ICF-based framework to simultaneously examine multiple impairment domains within a population-based setting. This allows for a comprehensive and function-oriented assessment of disability-related disparities.<br><br>Second, whereas most existing studies on cancer disparities by disability status have primarily examined screening uptake or treatment patterns, our study focuses on diagnostic pathways and emergency cancer diagnosis, which represents an underexplored dimension of cancer inequalities for people with disabilities. Reducing emergency cancer diagnoses has been recognised as a key |

| | |
|---|---|
| | public health priority, yet little is known on how it affects people with disability<br><br>These aspects have now been more clearly articulated in the revised Introduction and Discussion sections of the manuscript. |
| 2. The analyses demonstrate associations between impairment categories and emergency presentation; however, the mechanisms underlying these disparities remain largely speculative. The discussion proposes explanations such as diagnostic overshadowing or barriers to healthcare access, but these mechanisms are not directly evaluated in the analysis. The authors should acknowledge this limitation and avoid overinterpretation of causal pathways. | We thank the reviewer for this important comment. We agree that the mechanisms underlying the observed associations cannot be directly assessed with our study design. In response, we have revised the Discussion to better reflect this limitation and to avoid overinterpretation of potential causal pathways. In particular, we have reduced or removed more speculative explanations and focused on interpretations more directly supported by the data. We have explicitly acknowledged in the Limitations section: “The mechanisms underlying the observed associations could not be directly assessed, and further studies are needed to investigate the pathways linking impairment status to diagnostic routes and outcomes.”. |
| 3. Disability status is derived from administrative indicators mapped to ICF categories. This approach may not fully capture the severity or functional impact of impairments and could introduce misclassification bias. So, additional justification, validation references, or sensitivity analyses are needed for supporting this classification approach. | We thank the reviewer for this important and constructive comment. We agree that disability indicators derived from administrative data may not fully capture the severity or the full functional impact of impairments, and that some degree of misclassification is possible when functional status cannot be directly measured. As also specified in our response to Reviewer 1 - Point 2, we have now clarified this aspect more explicitly in the revised manuscript. Please see response to Reviewer 1 - Point 2 above for more details. |
| 4. Although the models adjust for several relevant variables, important potential confounders remain unmeasured, such as health behaviours, healthcare utilization patterns, frailty, and health literacy. These factors could influence both impairment status and the likelihood of emergency diagnosis, potentially affecting the estimated associations. | We agree that additional factors such as health behaviours, healthcare utilisation patterns, frailty and health literacy may influence both impairment status and the likelihood of emergency cancer diagnosis. Unfortunately, these variables are not available in the administrative health databases used in this study, and therefore residual confounding from unmeasured factors cannot be excluded. We have clarified this point in the Limitations section of the Discussion. Regarding frailty, we attempted to partially account for this dimension by adjusting all multivariable models for the number of chronic comorbidities. Albeit being different from frailty, the number of comorbidities can be considered as a proxy for the overall health status and possibly as an indication of clinical vulnerability. |

| | |
|---|---|
| | Potential confounding factors considered in our models are in line with previous international electronic health records studies on emergency cancer diagnosis, typically adjusting for age, sex, socioeconomic status, cancer site, stage and comorbidity burden, but not including behavioural or health literacy factors (McPhail S et al Lancet Oncology 2022; Zhou Y et al Nature Reviews Clinical Oncology 2017; Wallace D et al British Journal of Cancer 2014; Nilssen Y et al European Journal of Public Health. 2021).<br><br>Future studies integrating administrative data with clinical or survey-based information on health behaviours, frailty and healthcare utilisation would be valuable to clarify the mechanisms underlying the observed disparities. |
| 5. The observation that sensory impairments are associated with lower mortality in some analyses is interesting but insufficiently explained. The authors should provide a more detailed interpretation of this finding and discuss possible explanations such as detection bias, healthcare utilization patterns, or residual confounding. | We agree that the finding of lower mortality among patients with sensory impairments warrants further consideration. Several mechanisms may plausibly explain this association. To address the reviewer's comment, we have expanded the Discussion section to provide a more detailed interpretation of this finding and to acknowledge potential underlying mechanisms. In particular, we discuss how sensory impairments are relatively common in older populations and they may reflect less severe health deterioration compared with other impairment domains. At the same time, people with sensory impairments, compared to those without, might have more frequent contact with healthcare services and might be supported by carers or family members during clinical encounters, which may facilitate communication, care navigation, and timely diagnostic assessment. We additionally acknowledge that variability in impairment severity and residual confounding cannot be excluded.<br><br>We also highlight that, to our knowledge, previous studies have not specifically examined emergency cancer diagnosis by sensory impairment, and future research should explore these associations in more detail, including the potential mediating role of healthcare navigation, social support and accessibility of care. |
| 6. While the manuscript highlights disparities in cancer diagnosis among individuals with impairments, the discussion could more clearly articulate the clinical and health system implications of these findings. More specific recommendations regarding screening pathways, diagnostic processes, or healthcare accessibility for | We agree that the clinical and health system implications of our findings could be more clearly articulated. To address this comment, we have expanded the Discussion section (Implications for public health) to more explicitly outline the |

| | |
|---|---|
| disabled populations would strengthen the translational relevance of the study. | potential clinical and policy implications of the observed disparities in cancer diagnostic pathways among individuals with impairments. In particular, we now discuss how interventions could be implemented at multiple levels of the healthcare system. We reported: "At the clinical level, healthcare professionals should be supported with targeted training and practical tools to improve recognition of potential cancer symptoms in people with disabilities, including the use of structured symptom assessment approaches and clinical decision support, to reduce diagnostic overshadowing.<br><br>At the health system level, screening programmes and diagnostic services should ensure that invitations and information materials are provided in accessible formats (e.g., easy-read versions, large print, braille or digital formats compatible with assistive technologies), alongside physically accessible clinical environments and facilitation of caregiver or support person involvement during healthcare encounters. Patient navigation programmes and improved coordination between primary care, oncology services and disability support services may further facilitate timely referral and diagnostic assessment.<br><br>From a policy perspective, incorporating disability indicators into routine cancer monitoring may help identify vulnerable groups at higher risk of suboptimal diagnostic pathways and support the development of targeted interventions aimed at improving equity in cancer care. Given the ageing of the population and the increasing prevalence of disability and multimorbidity, strengthening disability-inclusive cancer services will be essential to ensure equitable access to timely diagnosis and treatment."<br><br>These additions were introduced to strengthen the translational relevance of the study and to more clearly highlight potential implications for clinical practice and health system organisation. |
| Minors<br>1. The rationale for focusing on one-year overall survival should be clarified, particularly given the varying survival trajectories across the cancer types included in the analysis.<br>2. Several stratified analyses are presented (e.g., by metastatic status and diagnostic route), but the clinical interpretation of these results could be more clearly explained in the text.<br>3. The potential impact of COVID-19 on diagnostic | 1. We thank the reviewer for this important comment. In this study, we focused on short-term (one-year) overall survival as Previous studies on cancer diagnosis following emergency presentation have consistently shown that this is more closely related to variations in stage at diagnosis, timeliness of diagnostic assessment, and access to initial treatment, whereas longer-term survival is more strongly influenced by disease progression and treatment-related factors (McPhail et al. (Lancet |

| | |
|---|---|
| pathways and healthcare access is only briefly discussed. Additional contextualization would improve interpretation.<br>4. The adjusted survival curves presented in the supplementary are somewhat complex. Clearer labeling or highlighting of key differences between impairment groups would improve readability. | Oncol, 2022). Short-term mortality is considered a possible indicator of diagnostic delay and our approach, was chosen to ensure comparability with recent national and international studies (Lancet Oncol, 2022) (Mitchell et al., Cancer Epidemiology, 2024Tørring et al., Br J Cancer, 2011; Nilssen et al., BMC Cancer, 2021,).<br><br>We have now clarified this rationale in the revised manuscript and added references to previous studies that have adopted a similar approach.<br><br>2. Please see responses 1,4 and 5 to reviewer 1.<br><br>3.We thank the reviewer for this helpful comment. In response, we have expanded both the Introduction and the Discussion to provide additional contextualisation of the potential impact of the COVID-19 pandemic on diagnostic pathways and healthcare access.<br><br>In the Introduction, we have added information on the specific context of Lombardy, one of the first European regions affected by the pandemic, highlighting the substantial strain on the healthcare system and the reorganisation of services, which may have influenced access to diagnostic pathways.<br><br>In the Discussion, we have further elaborated on how pandemic-related factors, including changes in healthcare-seeking behaviour and reduced access to diagnostic services, may have contributed to the increased risk of emergency presentation observed during this period, particularly among individuals with impairments.<br><br>These additions aim to improve the interpretation of our findings within the broader healthcare system context.<br><br>4. We thank the reviewer for this helpful comment. In response, we have revised the supplementary survival figures to improve their clarity and readability. In particular, we have refined the labelling and enhanced the visual presentation to better highlight the differences between impairment groups. |

**Sex and gender considerations**

Sex (biological attribute) was obtained from cancer registry data and included in the study design as an a priori covariate in all statistical analyses to account for potential confounding. Sex information was derived from routinely collected administrative and registry data and reflects legally recorded sex.

Descriptive analyses were disaggregated by sex, with results reported separately for males and females where appropriate.

Gender-related variables were not available in the data sources used for this study and could therefore not be assessed.

The study population included individuals of both sexes; therefore, findings are not restricted to a single sex and no modification to the title or abstract was required.

Sex was included as an adjustment variable in all multivariable models.

# Point-by-point response to the reviewers' comments

| | |
|---|---|
| Reviewer #1 (Remarks to the Author): The authors have responded well to my comments. | We thank Reviewer 1 for their positive assessment and for acknowledging that the comments have been adequately addressed. |
| Reviewer #2 (Remarks to the Author): The authors have substantially improved the manuscript and have addressed the concerns raised in the previous review. I recommend acceptance. | We also thank Reviewer 2 for their careful re-evaluation of the manuscript, their positive assessment of the revisions, and their recommendation for acceptance. |
